# Integrated lipidomics and proteomics network analysis highlights lipid and immunity pathways associated with Alzheimer’s disease

**DOI:** 10.1186/s40035-020-00215-0

**Published:** 2020-09-21

**Authors:** Jin Xu, Giulia Bankov, Min Kim, Asger Wretlind, Jodie Lord, Rebecca Green, Angela Hodges, Abdul Hye, Dag Aarsland, Latha Velayudhan, Richard J. B. Dobson, Petroula Proitsi, Cristina Legido-Quigley, on behalf of the AddNeuroMed Consortium

**Affiliations:** 1 Institute of Pharmaceutical Science, King’s College London, London, UK; 2 Institute of Psychiatry, Psychology & Neuroscience, King’s College London, London, UK; 3 Steno Diabetes Center Copenhagen, Gentofte, Denmark; 4 Center for Age-Related Medicine, Stavanger University Hospital, Stavanger, Norway

**Keywords:** Alzheimer’s disease, Dementia, Brain atrophy, sMRI, Rate of cognitive decline, Lipidomics, Proteomics, AD risk loci, WGCNA

## Abstract

**Background:**

There is an urgent need to understand the pathways and processes underlying Alzheimer’s disease (AD) for early diagnosis and development of effective treatments. This study was aimed to investigate Alzheimer’s dementia using an unsupervised lipid, protein and gene multi-omics integrative approach.

**Methods:**

A lipidomics dataset comprising 185 AD patients, 40 mild cognitive impairment (MCI) individuals and 185 controls, and two proteomics datasets (295 AD, 159 MCI and 197 controls) were used for weighted gene co-expression network analyses (WGCNA). Correlations of modules created within each modality with clinical AD diagnosis, brain atrophy measures and disease progression, as well as their correlations with each other, were analyzed. Gene ontology enrichment analysis was employed to examine the biological processes and molecular and cellular functions of protein modules associated with AD phenotypes. Lipid species were annotated in the lipid modules associated with AD phenotypes. The associations between established AD risk loci and the lipid/protein modules that showed high correlation with AD phenotypes were also explored.

**Results:**

Five of the 20 identified lipid modules and five of the 17 identified protein modules were correlated with clinical AD diagnosis, brain atrophy measures and disease progression. The lipid modules comprising phospholipids, triglycerides, sphingolipids and cholesterol esters were correlated with AD risk loci involved in immune response and lipid metabolism. The five protein modules involved in positive regulation of cytokine production, neutrophil-mediated immunity, and humoral immune responses were correlated with AD risk loci involved in immune and complement systems and in lipid metabolism (the APOE ε4 genotype).

**Conclusions:**

Modules of tightly regulated lipids and proteins, drivers in lipid homeostasis and innate immunity, are strongly associated with AD phenotypes.

## Background

There is an urgent need to further understand the pathways and processes underlying Alzheimer’s disease (AD) for early diagnosis and development of effective treatments. With the estimated number of patients suffering from dementia rising up to 115.4 million worldwide in 2050 [1], AD is undoubtedly one of the major healthcare challenges in the twenty-first century. Blood-based biomarkers serve as an easily accessible and minimally invasive screening tool to identify at-risk individuals for further investigation and monitoring or stratification in clinical trials. In addition, they can reveal molecular pathways leading to AD, providing new opportunities for drug development [2].

In the past decade, a large number of untargeted and targeted blood biomarker studies have revealed and replicated associations of proteins either individually or in combinations with AD and AD endophenotypes. These endophenotypes include brain atrophy, rate of cognitive decline (ROD) and amyloid burden [3, 4]. Although the majority of protein biomarkers have failed to survive further validations, several proteins, especially inflammatory proteins and proteins involved in the complement pathway, have been consistently associated with AD or AD endophenotypes, including complement C6 and C-C motif chemokine 15 [3].

More recently, a number of untargeted and targeted blood metabolomics studies have revealed the roles of lipids in AD [5–7]. Lipidomics aims to identify and quantify thousands of lipids. It is regarded as a subset of metabolomics [8, 9], reflecting functional networks of downstream changes of the genome, transcriptome and proteome [10], and bridging the phenotype–genotype gap due to their close association with cellular processes [11]. We have previously performed lipid phenotyping and identified a panel of 10 metabolites, which predicted an AD training dataset with 83% accuracy and a test dataset with 79% accuracy [12]. As in the case of proteins, results have not always been the same, but phosphatidylcholines (PCs), cholesteryl esters (ChEs), and triglycerides (TGs) have been consistently shown to be altered in mild cognitive impairment (MCI) and AD compared to controls [7, 12–14].

Most biomarker studies to date have been restricted to one modality (proteomics or metabolomics), and only a modest number have used systems biology approaches [15–17]. Network analysis methods provide a powerful tool to depict the disease-associated networks of highly connected molecules, which could be potential targets for AD investigation and treatment. A small number of blood and brain network studies using Weighted Gene (or Protein or Lipid) Correlation Network Analysis (WGCNA) have highlighted gene, protein and lipid pathways that are involved in the aetiology, initiation, and progression of AD [15, 18].

**Fig. 1:**
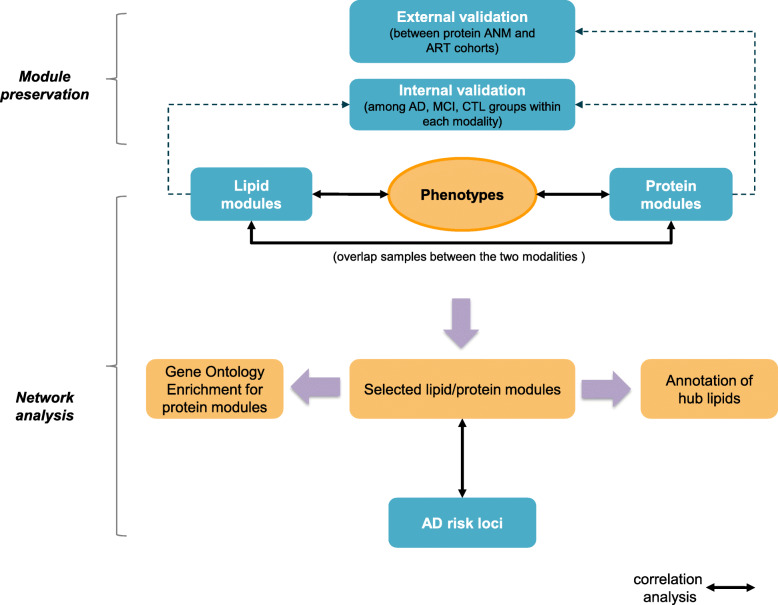
Study workflow. Protein and lipid modules were produced, and their preservation was investigated. An internal validation among AD, MCI and CTL groups was performed for the protein and lipid modules, and additional external validation of the protein modules was performed against the ART cohort. Correlation analyses among lipid modules, protein modules and phenotypes (clinical diagnosis, rate of cognitive decline, left and right hippocampal volumes, left and right entorhinal cortex volumes) were made separately and led to a selected number of modules. Gene ontology enrichment analysis was applied for selected protein modules, while the annotation of lipid species was conducted for selected lipid modules. The associations between lipid/protein modules and AD risk loci were also investigated

In the present study, we aimed to explore the role of blood lipids and proteins in AD at a systems level by performing an integrative multiscale network analysis and correlating the identified modules with AD diagnosis, brain atrophy and the ROD. As genome-wide association studies and meta-analyses have consistently implicated immunity and lipid processing in AD [19], we also integrated these networks with established AD risk loci (study design shown in Fig. 1).

## Methods

### Participants

Three datasets were employed in this study (Table 1). Briefly, dataset 1 contained proteomic data across 201 AD patients, 104 individuals with MCI and 97 controls from the EU-funded AddNeuroMed (ANM) study [20]. Dataset 2 consisted of lipidomic data across 185 AD, 40 MCI and 185 control individuals from the Maudsley and King’s Healthcare Partners Dementia Case Register (DCR) and the ANM study. The datasets 1 and 2 overlapped on 240 individuals, comprising 147 AD, 10 MCI and 83 controls. An additional proteomic dataset 3 from the Alzheimer’s Research Trust (ART) cohort [21] was included with 94 AD, 55 MCI and 100 control individuals.

**Table 1:** Sample demographics

Dataset	AD	MCI	Controls	Differences among three groups*
Proteomic dataset (dataset 1, *n* = 411)	*n* = 210	*n* = 104	*n* = 97	
Age (years), mean (SD)	77 (6.5)	75 (6.1)	72 (6.6)	*P* = 1.13e-08
Gender (female/male)	133/77	59/45	56/41	*P* = 0.4431
*APOE* ε4 allele (absence/presence)	89/115	58/46	65/32	*P* = 5.349e-04
ROD per year, mean (SD)^a^	−1.49 (1.26) (*n* = 127)	NA	NA	NA
Brain imaging	*n* = 53	*n* = 0	*n* = 67	
Whole brain volume, mean (SD)^b^	0.66 (0.073)	NA	0.69 (0.043)	*P* = 0.003184
Left hippocampal volume, mean (SD) ^b^	0.0018 (0.00037)	NA	0.0024 (0.00031)	*P* = 7.717e-15
Right hippocampal volume, mean (SD) ^b^	0.0019 (0.00041)	NA	0.0025 (0.00031)	*P* = 3.535e-13
Left entorhinal cortical volume, mean (SD) ^b^	0.00095 (0.00038)	NA	0.0012 (0.00024)	*P* = 1.433e-05
Right entorhinal cortical volume, mean (SD) ^b^	0.00092 (0.00034)	NA	0.0013 (0.00028)	*P* = 3.562e-08
Lipidomic dataset (dataset 2, *n* = 415)	*n* = 185	*n* = 40	*n* = 190	
Age (years), mean (SD)	77 (6.9)	75 (6.3)	79 (5.5)	*P* = 6.77e-05
Gender (female/male)	114/71	20/20	116/74	*P* = 0.377
*APOE* ε4 allele (absence/presence)	73/110	22/15	138/51	*P* = 8.725e-10
ROD per year, mean (SD)^a^	−1.50 (1.22) (*n* =144)	NA	NA	NA
Brain imaging results	*n* = 57	*n* = 8	*n* = 67	
Whole brain volume, mean (SD) ^b^	0.66 (0.071)	0.71 (0.041)	0.69 (0.098)	*P* = 0.0006595
Left hippocampal volume, mean (SD) ^b^	0.0018 (0.00038)	0.0021 (0.00031)	0.0024 (0.00060)	*P* = 1.763e-15
Right hippocampal volume, mean (SD) ^b^	0.0018 (0.00043)	0.0022 (0.00032)	0.0024 (0.00062)	*P* = 2.572e-13
Left entorhinal cortical volume, mean (SD) ^b^	0.00093 (0.00038)	0.0012 (0.00024)	0.0012 (0.00030)	*P* = 2.782e-06
Right entorhinal cortical volume, mean (SD) ^b^	0.00090 (0.00033)	0.0011 (0.00028)	0.0013 (0.00027)	*P* = 4.114e-09
Proteomic validation dataset (dataset 3, *n* = 249)	*n* = 94	*n* = 55	*n* = 100	
Age (years), mean (SD)	83 (6.2)	77 (4.5)	79 (7.0)	*P* = 2.02e-12
Gender (female/male)	72/22	35/20	47/53	*P* = 1.185e-04
*APOE* ε4 allele (absence/presence)	40/50	30/10	79/20	*P* = 8.204e-07

*AD* Alzheimer’s disease, *ROD* Rate of cognitive decline, *SD* Standard deviation, *NA* Not available

*Differences in the means/frequencies of clinical/demographic variables were tested using ANOVA, t test or χ2 test

^a^Data of the rate of cognitive decline were available for a subset of AD patients

^b^sMRI data were available from a subset of study participants

All individuals with AD met the criteria for probable AD according to the National Institute of Neurological and Communicative Disorders and Stroke and the Alzheimer’s Disease and Related Disorders Association criteria [22], and the fourth edition of the Diagnostic and Statistical Manual of Mental Disorders (DSM-IV) [23]. Subjects in the MCI group were mainly recruited from memory clinics, and scored 0.5 on the total Clinical Dementia Rating Scale (CDR) or 0.5 or 1 on the memory category of the CDR [24]. All MCI individuals reported memory problems, but showed no significant impairment in daily living according to the Petersen’s criteria of MCI [25]. Individuals in the control group showed no signs of cognitive impairment in the mini-mental state examination (MMSE) [26] or ADAS-cog assessment. Subjects were excluded from this study if they had any other significant psychiatric or neurological illness. All AD cases had an age-of-onset of at least 60 years, and the control and MCI individuals were 60 years or above at examination. Finally, AD diagnosis was confirmed by pathological examination in 5 AD patients from the DCR cohort and 15 AD patients from the ART cohort. Each individual was required to fast for 2 h before sample collection, and then 10 ml of blood was collected in tubes coated with sodium ethylenediaminetetraacetic acid to prevent clotting. The whole blood was centrifuged at 2000 g for 10 min under 4 °C to collect plasma and stored it at − 80 °C. All samples were centrifuged within approximately 2 h after collection.

### Proteomic and lipidomic analyses

The lipidomic and proteomic experiments have been described previously in detail [14, 27]. All plasma samples used for the lipidomic and proteomic analyses were collected at baseline visit. Overall, 1016 proteins with Uniprot ID were measured with a Slow Off-rate Modified Aptamer (SOMAmer)-based capture array called “SOMAscan” (SomaLogic, Inc). Lipidomics was performed by a Waters ACQUITY UPLC and XEVO QTOF system where 2216 lipid features were measured and included.

### Structural magnetic resonance imaging (sMRI)

Volumes of the whole brain, the hippocampi and the entorhinal cortices were obtained from subjects who had undergone sMRI (Table 1). Regions were normalized by intracranial volume [4]. The detailed information regarding data acquisition, pre-processing, and quality control assessment has been described elsewhere [28, 29]. Before analyses, sMRI measures were standardized to have a mean of 0 and a standard deviation (SD) of 1.

### Calculation of ROD

ROD was available from 127 AD patients in dataset 1 and 144 AD patients in dataset 2. The ROD was calculated based on longitudinal MMSE assessments [30], and only participants with at least three MMSE measures were included for linear mixed effect models [27]. Calculations were done separately for ANM and DCR due to the differences in assessment windows between the cohorts. After covariate adjustment, the slope coefficient obtained from the final model for each sample was then used as the ROD, defined as the change in MMSE score per day [27].

### Weighted lipid co-expression network analysis

Prior to network analysis, missing values were imputed using the k-nearest neighbour (KNN) imputation function from “impute” package within R version 3.6.1 [31]. Inverse normal transformation (quantile normalisation) was applied to normalize the lipidomics data (dataset 2). Principal component analysis (PCA) was conducted on the genotype data to extract principal components that represent the population structure. Each normalised lipid was then regressed against age, gender, batch and the first seven principal components. The lipid residuals were used in downstream analyses.

A weighted lipid co-expression network was built with R package “WGCNA” [32] using the inverse-normalized lipidomics residuals. Briefly, a thresholding power of 12 was chosen (as it was the smallest threshold that resulted in a scale-free *R*^2^ fit of 0.9) and the signed network was created by calculating the component-wise minimum values for topologic overlap (TO). Using 1 – TO (dissTOM) as the distance measure, lipid features were hierarchically clustered. Initial module assignments were determined by using a dynamic tree-cutting algorithm (“tree” method, cutHeight = 0.99, deepSplit = True, minModulesize = 30).

Before applying the network analysis to the whole lipidomics dataset, internal module preservation was applied to verify that module assignments were not affected by the diagnosis groups. This was conducted by splitting lipidomics dataset into three sub-datasets according to the clinical status (AD, MCI, or control). The module preservation analysis was applied for any two sub-datasets, assigning one as the reference dataset and the other as the test dataset.

The resulting 20 modules or clusters of co-expressed lipid features were selected by merging modules based on the clustering of module eigenlipids (MEs; or the 1st principal component of the module). The module membership (kME) quantifies how close a lipid is to a given module and can be measured by calculating the correlation between individual lipids and the ME. Lipids with high kME (top 10) in the module were informally referred to as top drivers [33].

The associations between the eigenlipids of 20 modules and six phenotypes including AD clinical diagnosis (AD vs controls), left and right hippocampal volumes, left and right entorhinal cortical volumes and the ROD, were investigated using Pearson correlation. Only modules associated with more than one phenotype and having at least one association passing Bonferroni correction threshold (*P* = 4.2e-04) were selected for further analysis. The relationship between lipid correlation with AD phenotypes and lipid module membership in selected modules was also investigated to examine the association of top module drivers with the examined phenotypes.

### Weighted protein co-expression network analysis

Similar pre-treatment approaches were applied to both ANM and ART proteomics datasets. After imputing missing values using KNN [31], log2 transformation was applied and values outside of 4 SDs of the mean were excluded. The log2-transformed and outlier-removed proteomics data were regressed against age, gender and the first seven PCs (for the ANM cohort). The resulting datasets were utilized for further analyses.

Two weighted protein co-expression networks were built for the above pre-treated datasets using WGCNA [32]. A thresholding power of 7 was chosen and the signed networks for both datasets were created following the same steps described in the “weighted lipid co-expression network” section, except for using a minimum module size of 17 [15]. For both proteomics datasets, 17 modules were built.

The internal preservation analyses as previously described were applied in each proteomics dataset to investigate whether the generated modules were preserved in AD patients, controls and MCI individuals. External validation was also conducted using dataset 1 as reference, dataset 3 as test and vice versa. Cross-tabulation analysis was then employed to investigate module overlap and correlations between the two datasets [34]. After validating the modules, Pearson’s correlation with Bonferroni correction (*P* = 4.9e-04) was applied to investigate the associations between the six phenotypes and the module eigenproteins for 17 modules in dataset 1. The relationship between individual protein correlations with AD phenotypes and protein module membership kME in selected modules was also explored as in the case of lipid modules.

### Associations between lipid and protein modules

The associations between five lipid and five protein modules that were associated with AD phenotypes at a Bonferroni corrected level were also analyzed with Pearson’s correlation coefficient in (i) all individuals (overlapping samples between ANM proteomics and lipidomics dataset, *n* = 240); (ii) only AD group; and (iii) only control group. The correlations of individual lipids to proteins in modules that passed Bonferroni correction (*P* = 2e-03) were further investigated at two different thresholds with correlation coefficient absolute values in the ranges of 0.1–1 and 0.2–1, respectively.

### Annotation of top drivers of the lipid modules and gene set enrichment analyses of protein modules

Lipid species annotation was performed in the selected lipid modules, i.e. those associated with at least two phenotypes. To examine the biological processes and the molecular and cellular functions of protein modules associated with the tested phenotypes, Gene Ontology (GO) enrichment analysis against KEGG and Reactome pathways and Over-representation analysis (ORA) were performed using WebGestalt (WEB-based Gene SeT AnaLysis Toolkit) [35]. The genome database was used as the background/reference set. Z scores determined the over-representation of ontologies in a module and one-tailed Fisher’s exact tests (Benjamini-Hochberg FDR corrected) were used to assess the significance of the Z score [36]. A minimum of five genes per ontology were used as filters prior to pruning the ontologies and the FDR significant (*q* < 0.05) or top 10 categories were selected.

### Lipid and protein association with AD genetic variants

Genetic data from the blood samples of the ANM-DCR studies were obtained using the Illumina 610-Quad chip in three different batches as previously described [37]. After quality control using PLINK [38], genetic data were mapped to the “build37” reference genome and imputation was performed using IMPUTE2 [39]. Individuals with > 10% missingness and variants with > 5% missingness were excluded. The remaining variants were filtered using a minor allele frequency threshold of 5%, leaving ~ 4.5 M genetic variants for each dataset.

Linear regression was used to investigate associations of 34 established AD risk variants [19, 40–43] (Table S1) with the five lipid and five protein modules (residuals) associated with the six tested phenotypes. Genetic variants associated with AD at *P* < 5e-08 in genome-wide association and meta-analyses (GWAMA) studies published until December 2019 were used. Variants from the study by Kunkle et al. [19] were primarily used as this was the largest GWAMA study with clinical AD diagnosis. Additional variants that were not identified by Kunkle et al. but reached genome-wide significance in four large GWA and GWAMA studies [40–43] were also included. When a risk variant was not available in our dataset, a proxy in high linkage disequilibrium (*r*^2^ ≥ 0.8) was used. The Bonferroni corrected *P* < 1.5e-04 was used to account for the number of variants (*n* = 34) and phenotypes (*n* = 10) tested; however, nominal associations at *P* < 0.05 were also investigated.

## Results

### Demographics of participants

There were no differences in gender among the three diagnostic groups in both datasets 1 and 2 (Table 1). The AD participants had an older age than MCI and control participants in datasets 1 and 3. There were significantly more *APOE* ε4 carriers in the AD group than in the other two groups in all the three datasets (all *P* < 0.01). MRI analyses showed lower hippocampal volumes (left and right) and entorhinal cortical volumes in the AD participants (all *P* < 0.01).

### Module preservation

To assess the reliability and reproducibility of the established modules and to investigate whether the modules were preserved among the AD patients, individuals with MCI and healthy controls, module preservation analysis was applied. Most of the lipid and protein modules showed medium-to-high preservation among AD cases, individuals with MCI and controls. Therefore, WGCNA was applied to all three groups (Figs. S1 and S2). External validation was conducted for the ANM proteomics dataset versus the ART cohort (Fig. S3). Overall, there was good agreement between the ANM and ART cohort module assignments. Five ANM modules (black, blue, grey60, red and lightyellow) were well preserved in ART (black, green yellow, turquoise, blue and lightgreen, respectively) (Fig. S3). Conversely, the ANM module midnightblue appeared not to be preserved in ART networks since most of its proteins were classified as unassigned (grey colour). The rest of the modules in the ANM network appeared to show medium preservation and split into one or more modules in the ART network. For example, lightcyan module in the ANM network showed high preservation with salmon and lightyellow modules in the ART network (Fig. S3).

Weighted lipid correlation network analysis identified 17 modules of co-regulated lipids ranging from 36 to 328 lipids in each module (Fig. S4A). Weighted protein correlation network analysis identified 17 modules of co-regulated proteins ranging from 20 to 159 proteins in each module (Fig. S4B).

### Association of lipid modules with AD phenotypes

**Fig. 2:**
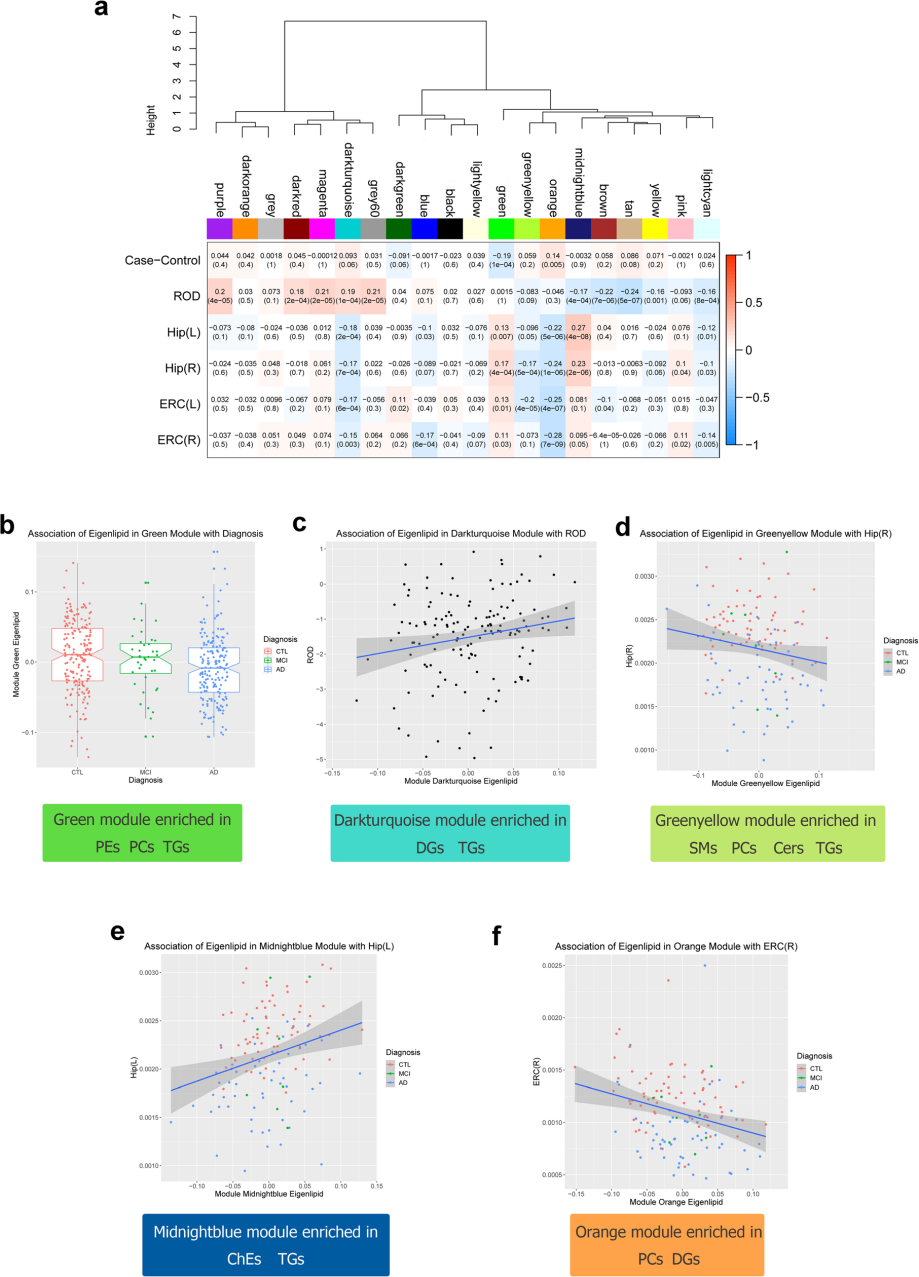
Correlations between lipid modules and AD phenotypes. **a** Lipid modules were clustered to assess module relatedness based on the correlations of lipid network eigenlipids (top). Heat map showing the correlation between lipid module eigenlipids and phenotypes (bottom). **b** Association of eigenlipid with diagnosis and main lipid species in the green module. **c** Association of eigenlipid with ROD and main lipid species in the darkturquoise module. **d** Association of eigenlipid with the left entorhinal cortical volume and main lipid species in the greenyellow module. **e** Association of eigenlipid with the left hippocampal volume and main lipid species in the midnightblue module. **f** Association of eigenlipid with the right entorhinal cortical volume and main lipid species in the orange module

After Bonferroni correction, 11 lipid modules were associated with at least one trait (Fig. 2a). Most associations were observed with ROD, where five modules showed positive associations and three modules showed negative associations. In addition, one module (green) was reduced in AD versus controls and five modules showed associations with brain atrophy, including two modules associated with less brain atrophy and three with increased brain atrophy. Of the eight modules correlated with ROD, only three were also associated with AD diagnosis or brain atrophy. Overall, there were five modules associated with at least two phenotypes. These modules included the darkturquoise module, which was associated with faster ROD and greater atrophy; green module, which was decreased in AD and was also associated with less atrophy; greenyellow module, which was associated with greater atrophy; midnightblue module, which was associated with slower ROD and less atrophy in the hippocampus; and orange module, which was associated with greater brain atrophy and was increased in AD, though the association with AD did not survive multiple testing (Fig. 2b–f).

We further investigated the five modules associated with at least two phenotypes. The top drivers for each of the five modules based on their kME are listed in Table S2. Annotations for all lipids in each module revealed that the green module was enriched in phosphatidylethanolamines (PEs), PCs and TGs; the orange module was enriched in PCs and diacylglycerides (DGs); the greenyellow module was enriched in sphingomyelins (SMs), PCs, ceramides (Cers) and TGs; the darkturquoise module was enriched in TGs and DGs; and the midnightblue module was enriched in TGs and ChEs (Table S3). We subsequently observed strong associations between the module membership of the lipids in the five modules and the lipid-phenotype correlations, indicating that lipids with high kME (i.e. top module drivers/hub lipids) were also associated with the respective AD phenotypes. Figure 2b displays the association of the green module with clinical diagnosis and Figure S5A shows the association of lipid kME in the green module against the lipid-diagnosis correlation. The rest of the associations between lipid modules and phenotypes that passed Bonferroni correction are presented in Supplementary Figs. S6 and S7. The lipid module memberships in the five selected modules and the lipid-phenotype correlations that passed Bonferroni correction are presented in Fig. S8.

### Association of protein modules with AD phenotypes

**Fig. 3:**
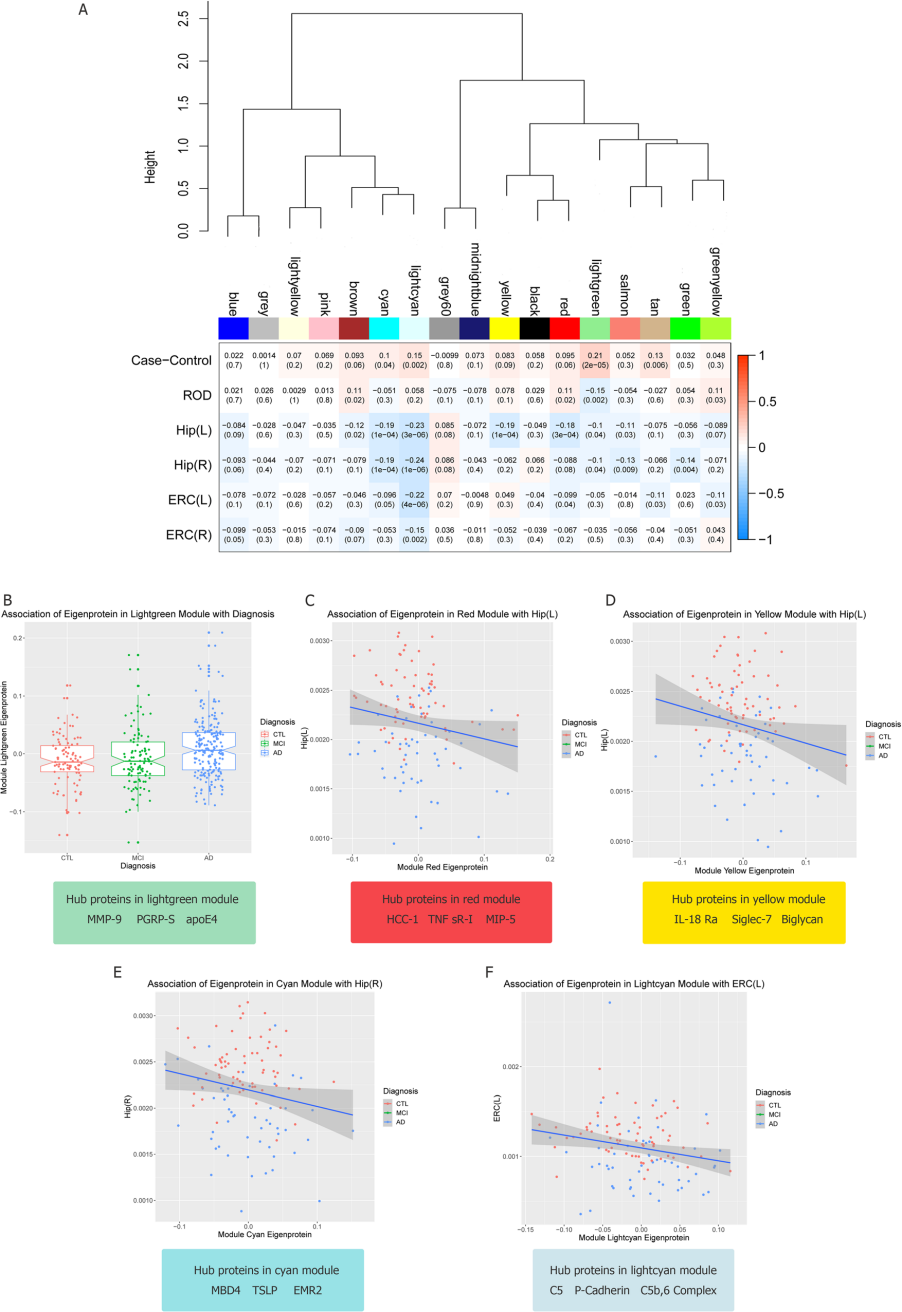
Correlations between protein modules and AD phenotypes. **a** Protein modules were clustered to assess module relatedness based on the correlations of protein network eigenprotein (top). Heat map showing the correlation between protein module eigenproteins and phenotypes (bottom). **b** Association of eigenprotein with diagnosis and hub proteins in the lightgreen module. **c** Association of eigenprotein with the left hippocampal volume and hub proteins in the red module. **d** Association of eigenprotein with the left hippocampal volume and hub proteins in the yellow module. **e** Association of eigenprotein with the right hippocampal volume and hub proteins in the cyan module. **f** Association of eigenprotein with the left entorhinal cortical volume and hub proteins in the lightcyan module

After Bonferroni correction, five protein modules were shown to be associated with at least one trait (Fig. 3a). Four of the five modules (yellow, red, cyan and lightcyan) were associated with increased brain atrophy and the eigenprotein in one module (lightgreen) was increased in AD patients compared to controls. Most associations were observed for the left hippocampal volume. Although some modules were associated with more than one phenotype, most of these associations did not pass multiple testing corrections (Fig. 3b-f).

The top drivers for each of the five protein modules, based on their kME, are displayed in Table S4. Overall, the protein module membership was lower compared to that of lipids. The top drivers in the five modules included many AD protein candidates, some of which have been associated with AD in the same cohort. ApoE (apoE3 and apoE4), one of these top drivers in the lightgreen module, is involved in complement cascade and growth factors. The results of GO over-representation analyses of proteins in the five modules associated with AD phenotypes are presented in Table S5. The biological processes that passed the Benjamini-Hochberg (BH) correction for each protein module are shown in the directed acyclic graph (DAG) in Figure S9. Biological GO terms that passed BH correction for the lightgreen module included neutrophil-mediated immunity, granulocyte activation, STAT cascade and regulation of inflammatory response; biological GO terms for the cyan module included positive regulation of cytokine production and mast cell activation; biological GO terms for the lightcyan module included protein activation cascade, insulin-like growth factor receptor signaling pathway and regulation of plasma lipoprotein particle levels; and biological GO terms for the red and yellow modules included humoral immune response. For KEGG pathway analysis, GO terms like complement and coagulation cascades which passed BH correction in the lightcyan and yellow modules, were also included in the cyan module; the JAK-STAT signalling pathway was listed as one of the top 10 pathways in the lightgreen module and the cyan module; the MAPK signalling pathway was highlighted in the red module after BH correction.

When we plotted kME of the proteins in the five modules versus the protein-phenotype correlations, the correlations were not as strong as in the case of lipids, except for the lightgreen module with AD diagnosis (Fig. 3b). Figure S5B illustrates the association of protein kME in the lightgreen module against the protein-diagnosis correlation. The rest of the associations between protein modules and phenotypes that passed Bonferroni correction are presented in Figure S10. The protein module membership was plotted against the protein-phenotype correlations that passed Bonferroni correction (Fig. S11).

### Association between lipid modules and protein modules

**Fig. 4:**
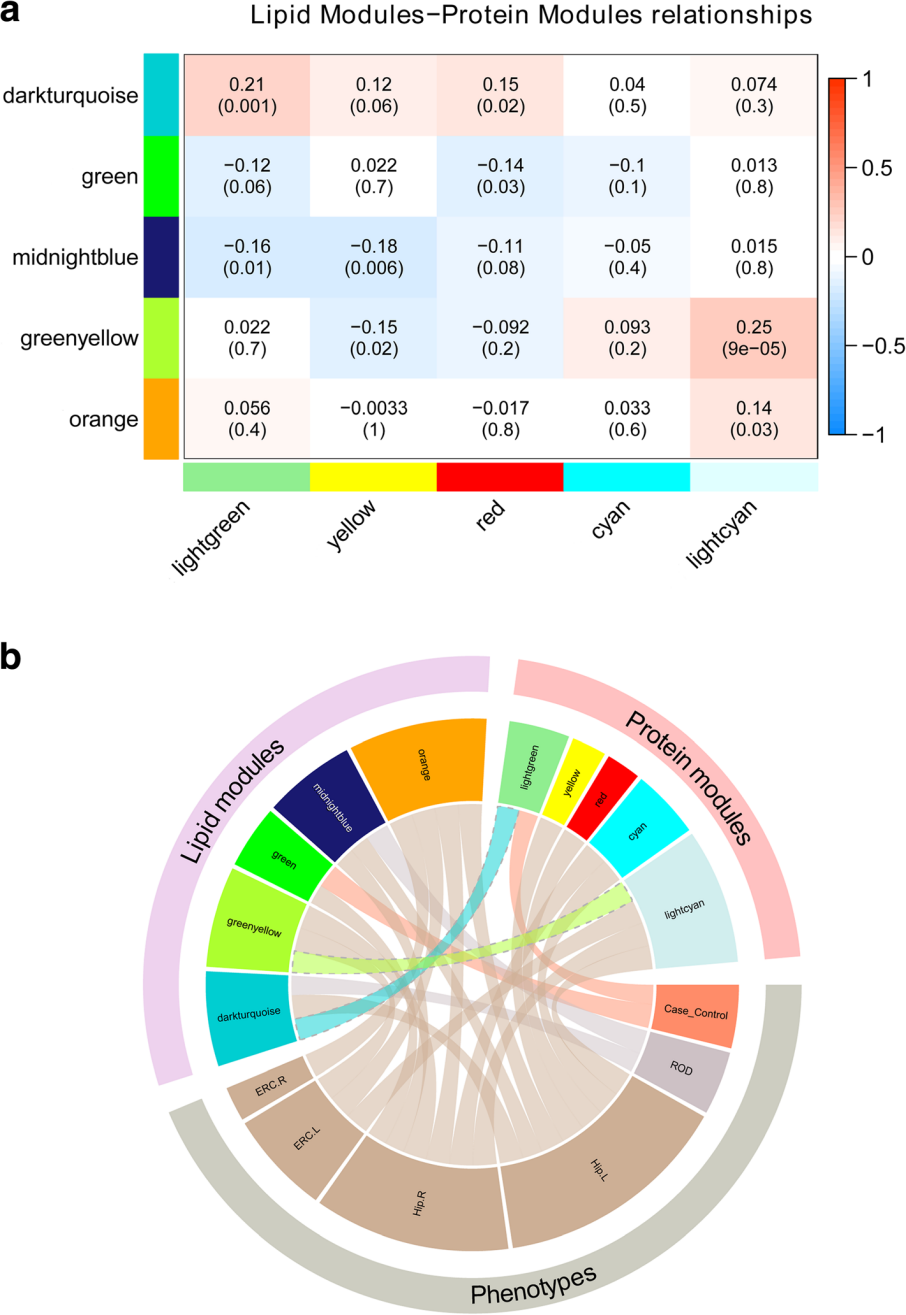
Associations among protein networks and lipid networks and phenotypes. **a** Heat map showing the Pearson correlations and *P* values (in bracket) between 5 lipid modules (rows) and 5 protein modules (columns) associated with phenotypes. **b** Circus plot showing the correlations among 5 lipid modules, 5 protein modules and 6 phenotypes

The relationship between lipid modules and protein modules associated with AD phenotypes was also investigated. The greenyellow lipid module was positively correlated with the lightcyan protein module after correcting for multiple testing (Fig. 4a). The darkturquoise lipid module was positively correlated with the lightgreen protein module. We further investigated the correlations between lipid and protein modules in AD and controls separately, but not in the MCI group due to the small size of MCI participants in both modalities. Interestingly, the high correlations between lipids and protein modules were mainly observed in AD patients rather than in controls (Fig. S12). The summary of associations among five lipid modules, five protein modules and six phenotypes is illustrated by the circus plot in Fig. 4b. It can be seen that the lipid greenyellow module and the protein lightcyan module, which were correlated with each other, were also associated with greater brain atrophy (both negatively correlated with the right hippocampal volume and the left entorhinal cortical volume).

The correlations between individual lipids in the greenyellow module and proteins in the lightcyan module, as well as between lipids in the darkturquoise module and proteins in the lightgreen module, were further investigated at two different thresholds (Figs. S13 and 14). To obtain an overview, the first threshold of correlation coefficient within the range of [− 1, − 0.1] & [0.1, 1] was applied, where some evident correlations between a number of proteins and lipids features were observed. To assess the strength of correlations, the second more strict threshold of [− 1, − 0.2] & [0.2, 1] was employed. The ApoB, PAFAH, and P-cadherin in the protein lightcyan module were strongly correlated with a group of lipids including DG (38:2) and some top drivers as listed in Table S2 from the greenyellow module. Similarly, both apoE3 and apoE4 in the protein lightgreen module showed strong correlation with some top lipid drivers in the lipid darkturquoise module.

### Association between AD genetic variants and selected lipid modules/protein modules

The five lipid modules (darkturquoise, green, greenyellow, midnightblue and orange modules) associated with more than 1 AD phenotype, and all five protein modules (lightgreen, red, yellow, cyan and lightcyan modules) associated with AD phenotypes were further analyzed for the associations with 34 AD genetic variants.

**Fig. 5:**
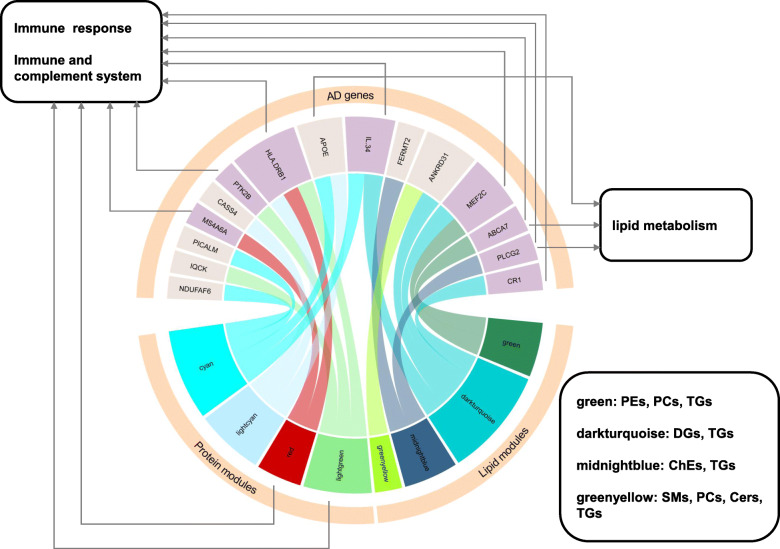
Summary of lipid module/protein module associations with AD risk loci. AD genetic variants *IL34, MEF2C*, *ABCA7*, *PLCG2* and *CR1* were correlated with the green, darkturquoise, and midnightblue lipid modules, which had established functions of immune response and inflammation, cholesterol metabolism, lipid transport, and immune and complements systems. Two protein modules, cyan and red, were linked with immune response through over-representation analysis, while AD genetic variants *MS4A6A*, *HLA-DLB1* and *IL34*, which were correlated with protein modules, also had immune response functions

Four of the five lipid modules that are enriched in PEs, PCs, TGs, DGs and CEs showed association with six genetic variants including *IL34*, *FERMT2*, *MEF2C*, *ABCA7*, *PLCG2*, *ANKRD31* and *CR1*. In addition, four out of five protein modules demonstrated association with nine genetic variants (*IL34*, *APOE*, *HLA-DRB1*, *PTK2B*, *CASS4*, *MS4A6A*, *PICALM*, *IQCK* and *NDUFAF6*). However, all of these associations were at the *P* < 0.05 level and none of them passed correction for multiple testing with Bonferroni corrected threshold of *P* < 1.5e-04 (listed in Table S6). For the *APOE* gene, both rs429358 and the *APOE* ε4 genotype were nominally associated with increased levels of cyan and lightcyan protein modules. The summarized associations between lipid modules and AD risk loci, as well as between protein modules and AD risk loci are shown in Fig. 5.

## Discussion

In this study, we employed a multi-omic network approach on data available for a total of 586 participants: 415 with lipidomics data, 411 with proteomics data, and 249 with proteomics validation data from three prospective cohorts. Overall, we identified modules of tightly regulated lipids and proteins that were strongly associated with AD phenotypes. The lipid modules had more abundant associations, mostly with ROD, followed by the brain volume. Conversely, most protein modules demonstrated associations with brain atrophy, particularly with the hippocampal volume. A number of lipid and protein clusters were also observed to be correlated with each other, especially in AD patients. Finally, the AD-associated modules demonstrated modest associations with AD genes involved in lipid and immune processes. Individual lipids/proteins were grouped into clusters/modules using network analysis and the associations with relevant clinical parameters were investigated [44].

The lipid co-expression network analysis revealed strong correlations of 11 lipid modules with AD diagnosis, ROD and brain atrophy measurements. These modules were preserved amongst AD, MCI and control groups; thus, the clustering of lipid features was not affected by clinical diagnosis. Not all modules showed associations across the spectrum of tested phenotypes with some being restricted to brain atrophy or ROD, suggesting distinct biological mechanisms. In addition, the five lipid modules associated with more than one phenotype were enriched in PCs, TGs and ChEs, which have been shown to be linked with AD in our studies including analyses on this cohort [12–14]. For example, the green module, which is enriched in TGs, is reduced in MCI and healthy control groups compared to AD cases, and positively correlated with entorhinal and hippocampal volumes. We also observed that a lipid feature annotated as TG (60:11), with a mass-to-charge ratio of 970.79, was previously associated with AD diagnosis and hippocampal volume by machine learning on overlapping samples [14]. This was one of the top drivers in the lipid green module, highlighting the importance of big TGs as possible drivers in AD processes. A recent study using the Alzheimer’s Disease Neuroimaging Initiative (ADNI) cohort highlighted the association of TGs, especially poly-unsaturated fatty acid-containing TGs, with AD and AD biomarkers [45]. TGs are also associated with Aβ, and longitudinal studies have shown that midlife TGs are associated with amyloidosis and tau pathology 20 years later in cognitively healthy individuals [46].

The protein modules identified were highly preserved through internal validation, as well as in an independent proteomic cohort. Five of these modules were strongly associated with AD phenotypes. With the development of analytical platforms, increasing numbers of studies have attempted to find AD biomarkers in blood. From the nominated biomarkers, only some have been replicated. The reason for such imbalance might be caused by the heterogeneity of the disease itself as well as the complexity of blood analysis [3]. Meta-analyses can help translate discovery findings to reproducible and useful biomarkers. In this study, we observed that some of the top drivers of the 5 AD-associated modules (Table S4) were linked to AD pathology in regression and random forest analyses on the same cohort; these included C-C motif chemokines from the red module associated with left hippocampal atrophy; carbonic anhydrase III and peptidoglycan recognition protein 1 from the lightgreen module correlated with AD diagnosis [27]. In addition, matrix metallopeptidase 9 [47, 48] and ApoE [48–52] from the lightgreen module, and complement component C6 [53] from the lightcyan module, had been highlighted in other AD biomarker studies. These findings underline the importance of these proteins as drivers of networks and pathways associated with AD.

Over-representation analysis of the 5 AD-related protein modules highlighted immune responses as the main biological process. KEGG pathways also highlighted the involvement of the complement, cytokine, MAPK signalling and the JAK-STAK pathways. Further, we observed correlations between lipid and protein modules that were associated with AD phenotypes, such as the association between the lightcyan protein module (enriched in complement proteins) and the greenyellow lipid module, suggesting important relationships and pathways that warrant further investigation. One of the GO biological terms revealed for the lightcyan protein module was the regulation of plasma lipoprotein particle levels. Molecular and cellular processes also highlighted the protein-lipid complex and phospholipid binding and KEGG pathways including glycophospholipid biosynthesis (although these were associated at FDR < 0.1). We also observed that the correlation between lipids and proteins was more pronounced in AD patients than in controls. This result is of great interest as it highlights that in addition to the changes in the level of each modality in AD (and AD endophenotypes), their interplay seems to be also disease-specific. These findings warrant further investigation.

Finally, we observed that the AD risk loci *CR1*, *PLCG2*, *MEF2C*, *IL34* and *ABCA7* were associated with several AD-related lipid modules. These loci are involved in immune and complement system [54], lipid metabolism and immune system [55], immune response and inflammation [41, 56], cytokine signalling in immune system [57], cholesterol/lipid metabolism, and immune and complement systems [58]. We also found that the protein modules highlighting immune response as the main biological process were nominally associated with variations of the *PTK2B*, *IL34*, *HLA-DRB1* and *MS4A6A* (gene cluster) risk loci, which are all related to immune response and the immune and complement systems [41, 58, 59]. Finally, the lightcyan and cyan modules were nominally associated with *APOE* ε4. As mentioned above, one of the GO biological terms for lightcyan is the regulation of plasma lipoprotein particle levels, and this was additionally associated with the lipid greenyellow module. However, none of these associations passed correction for multiple testing, which might be due to the small sample size of the study. Therefore, these findings merit further investigation, and need to be replicated in larger independent cohorts with available omics information. In addition, approaches such as Mendelian Randomization will help to probe the causal links between lipids, proteins and AD.

A limitation of our study is that the sample size was small. Further replication is therefore warranted, especially for genetic association analyses. In addition, we were not able to identify a good proxy for two genetic variants in two genes (*BIN1*, proxy *r*^2^ = 0.5; *INPP5D*, proxy *r*^2^ = 0.75; Table S1). Although we had overlapping lipidomics and proteomics data, these were available for only a subset of the study participants. Further, although we had information for ROD, this calculation was based on the MMSE, which is only one measure of cognition. Another limitation of the present study is that the MCI cohort included did not convert to AD within 1–3 year (stable MCIs) and therefore, we did not know whether these features are associated with the conversion from MCI to AD. Indeed, the module profile of the MCI group was closer to that of controls (Figs. 2b and 3b). In addition, the neuroimaging data of MCI participants were only available from 8 individuals. Our study was also limited inherently by the AD case-control design, in which some of the elderly controls may have already carried pathology, and that some of the clinically diagnosed AD may be pathologically non-AD dementias. However, although there was no information on β-amyloid or tau biomarkers (A/T biomarkers), this study provided neuroimaging data (an N biomarker, neuroimaging to measure brain atrophy) including volumes of hippocampi and entorhinal cortices, as well as data on the ROD in AD, which captured different stages of disease pathology including the early preclinical stages. Additionally, diagnosis was confirmed by pathologic examination for 20 AD cases. Finally, through the longitudinal nature of these cohorts, we know that all AD patients, individuals with MCI and healthy controls employed in our analysis maintained their baseline diagnosis throughout the follow-up period, which lasted between 1 and 5 years.

## Conclusions

Our study highlights that the interpretation of multi-omics data such as lipidomics, proteomics and genomics can be boosted by deploying systems biology approaches. Our integrative approach highlights tightly regulated and inter-connected networks of lipids and proteins associated with AD and AD phenotypes, with lipid and immunity pathways at the centre.

## Supplementary information

**Additional file 1: Table S1.** Genetic variants used for associations with lipid and protein modules. **Table S2.** Top 10 drivers in five selected lipid modules. **Table S3.** Selected lipid modules and annotation.** Table S4.** Top 10 drivers in five selected protein modules. **Table S5.** Summary of gene set enrichment analyses and gene ontology enrichment analysis of protein modules. **Table S6.** Correlations between lipid modules/protein modules and AD genetic variants. **Figure S1.** Preservation summary plots for AD, MCI and control sub datasets in lipidomics dataset. **Figure S2.** Preservation summary plots for AD, MCI and control sub datasets in ANM proteomics dataset. **Figure S3.** Dendrogram and cross-tabulation based comparison of modules in ANM and ART protein cohort networks. **Figure S4.** A. Cluster dendrogram of weighted lipid correlation network analysis and the number of lipid features in each module; B. Cluster dendrogram of weighted protein correlation network analysis and the number of proteins in each module. **Figure S5.** Scatter plots of module membership versus lipids/proteins-diagnosis correlation. **Figure S6.** Scatter plots of eigenlipids correlation with ROD. **Figure S7.** Scatter plots of eigenlipids correlation with brain atrophy measures. **Figure S8.** Scatter plots of lipid module membership versus lipids-phenotypes correlation. **Figure S9.** DAGs summarize biological processes in five protein modules. **Figure S10.** Scatter plots of eigenproteins correlation with brain atrophy measures. **Figure S11.** Scatter plots of protein module membership versus proteins-phenotypes correlation. **Figure S12.** Scatter plots of protein module membership versus proteins-phenotypes correlation. **Figure S13.** Correlation networks for lipid greenyellow module and protein lightcyan module. **Figure S14.** Correlation networks for lipid darkturquoise module and protein lightgreen module.

## Data Availability

The data that support the findings of this study are available from AddNeuroMed/ART/DCR consortiums but restrictions apply to the availability of these data, which were used under license for the current study, and so are not publicly available. Data are however available from the authors upon reasonable request and with permission of AddNeuroMed/ART/DCR consortiums.

## Abbreviations

AD: Alzheimer’s disease
WGCNA: Weighted Gene Co-expression Network Analyses
PC: Phosphatidylcholine
ChE: Cholesteryl ester
TG: Triglyceride
MCI: Mild cognitive impairment
ANM: AddNeuroMed
DCR: Dementia Case Register
ART: Alzheimer’s Research Trust
MMSE: Mini mental state examination
SOMAmer: Slow Off-rate Modified Aptamer
sMRI: Structural magnetic resonance imaging
ROD: Rate of cognitive decline
KNN: K-nearest neighbour
PCA: Principal component analysis
GO: Gene Ontology
ORA: Over-representation analysis
DG: Diacylglyceride
SM: Sphingomyelin
Cer: Ceramide
kME: Module membership
BH: Benjamini-Hochberg
