# Supplementary material for: Integrated lipidomics and proteomics network analysis highlights lipid and immunity pathways associated with Alzheimer’s disease
Source: Transl Neurodegener. 2020 Sep 21;9:36. doi: 10.1186/s40035-020-00215-0 (PMC7504646; doi:10.1186/s40035-020-00215-0)
Supplement: Supplementary file 1 — Additional file 1: Table S1. Genetic variants used for associations with lipid and protein modules. Table S2. Top 10 drivers in five selected lipid modules. Table S3. Selected lipid modules and annotation. Table S4. Top 10 drivers in five selected protein modules. Table S5. Summary of gene set enrichment analyses and gene ontology enrichment analysis of protein modules. Table S6. Correlations between lipid modules/protein modules and AD genetic variants. Figure S1. Preservation summary plots for AD, MCI and control sub datasets in lipidomics dataset. Figure S2. Preservation summary plots for AD, MCI and control sub datasets in ANM proteomics dataset. Figure S3. Dendrogram and cross-tabulation based comparison of modules in ANM and ART protein cohort networks. Figure S4. A. Cluster dendrogram of weighted lipid correlation network analysis and the number of lipid features in each module; B. Cluster dendrogram of weighted protein correlation network analysis and the number of proteins in each module. Figure S5. Scatter plots of module membership versus lipids/proteins-diagnosis correlation. Figure S6. Scatter plots of eigenlipids correlation with ROD. Figure S7. Scatter plots of eigenlipids correlation with brain atrophy measures. Figure S8. Scatter plots of lipid module membership versus lipids-phenotypes correlation. Figure S9. DAGs summarize biological processes in five protein modules. Figure S10. Scatter plots of eigenproteins correlation with brain atrophy measures. Figure S11. Scatter plots of protein module membership versus proteins-phenotypes correlation. Figure S12. Scatter plots of protein module membership versus proteins-phenotypes correlation. Figure S13. Correlation networks for lipid greenyellow module and protein lightcyan module. Figure S14. Correlation networks for lipid darkturquoise module and protein lightgreen module. [file 40035_2020_215_MOESM1_ESM.docx]

Supplementary materials

**Integrated lipidomics and proteomics network analysis highlights lipid and immunity pathways associated with Alzheimer’s disease**

Jin Xu^a,b^, Giulia Bankov^b^, Min Kim^c^, Asger Wretlind^c^, Jodie Lord^b^, Rebecca Green^b^, Angela Hodges^b^, Abdul Hye^b^, Dag Aarsland^b^, Latha Velayudhan^b^, Richard J.B. Dobson^b,*^, Petroula Proitsi^b,^*, Cristina Legido-Quigley^a,c,^* and on behalf of the AddNeuroMed Consortium

^a^Institute of Pharmaceutical Science, King’s College London, United Kingdom.

^b^Institute of Psychiatry, Psychology and Neuroscience, King’s College London, United Kingdom.

^c^Steno Diabetes Centre, Copenhagen, Denmark

*Corresponding authors:

Tel.: 004402078480924; Fax: 004402078480866; E-mail address: richard.j.dobson@kcl.ac.uk (R.J.B.D)

Tel.: 00442078480630; Fax: 004402078485914; E-mail address: petroula.proitsi@kcl.ac.uk (P.P)

Tel.: 004530913083; E-mail address: cristina.legido.quigley@regionh.dk (C.L.Q)

**Table S1. Genetic variants used for associations with lipid and protein modules.**

| **SNP** | **Chromosome** | **Base**  **position** | **GWS Gene**  **/Locus** | **A1/A2** | **A1 freq** | **Used** | **Reference**  **study** |
| --- | --- | --- | --- | --- | --- | --- | --- |
| rs4575098 | 1 | 161155392 | ADAMTS4 | A/G | 0.24 | Yes | [1] |
| rs4844610 | 1 | 207802552 | CR1 | A/C | 0.187 | Yes | [2] |
| rs6733839* | 2 | 127892810 | BIN1 | T/C | 0.407 | Yes | [2] |
| rs10933431** | 2 | 233981912 | INPP5D | G/C | 0.223 | Yes | [2] |
| rs6448453 | 4 | 11026028 | CLNK | A/G | 0.252 | Yes | [1] |
| rs4704171 | 5 | 74368254 | ANKRD31 | C/T | 0.123 | Yes | [3] |
| rs190982 | 5 | 88223420 | MEF2C | A/G | 0.39 | Yes | [4] |
| rs9271058 | 6 | 32575406 | HLA-DRB1 | A/T | 0.27 | Yes | [2] |
| rs9381040 | 6 | 41154650 | TREML2 | C/T | 0.281 | Yes | [5] |
| rs4723711 | 7 | 37844263 | NME8 | A/T | 0.356 | Yes | [4] |
| rs12539172 | 7 | 100091795 | NYAP1 | T/C | 0.303 | Yes | [2] |
| rs10808026 | 7 | 143099133 | EPHA1 | A/C | 0.199 | Yes | [2] |
| rs73223431 | 8 | 27219987 | PTK2B | T/C | 0.367 | Yes | [2] |
| rs9331896*** | 8 | 27467686 | CLU | C/T | 0.387 | Yes | [2] |
| rs10098778 | 8 | 95992020 | 95992020 | C/T | 0.47 | Yes | [3] |
| rs7920721 | 10 | 11720308 | ECDH3 | G/A | 0.389 | Yes | [2] |
| rs3740688 | 11 | 47380340 | SPI1 | G/T | 0.448 | Yes | [2] |
| rs7933202 | 11 | 59936926 | MS4A6A | C/A | 0.391 | Yes | [2] |
| rs3851179 | 11 | 85868640 | PICALM | T/C | 0.356 | Yes | [2] |
| rs17125924 | 14 | 53391680 | FERMT2 | G/A | 0.093 | Yes | [2] |
| rs12881735 | 14 | 92932828 | SLC24A4 | C/T | 0.221 | Yes | [2] |
| rs76523702 | 15 | 51002342 | SPPL2A | T/C | 0.2 | Yes | [5] |
| rs593742 | 15 | 59045774 | ADAM10 | G/A | 0.295 | Yes | [2] |
| rs4985556 | 16 | 8177320 | IL34 | A/G | 0.111 | Yes | [5] |
| rs7185636 | 16 | 19808163 | IQCK | C/T | 0.18 | Yes | [2] |
| rs59735493 | 16 | 31133100 | KAT8 | A/G | 0.3 | Yes | [1] |
| rs12444183 | 16 | 81942028 | PLCG2 | A/G | 0.39 | Yes | [5] |
| rs113260531 | 17 | 5138980 | SCIMP | A/G | 0.12 | Yes | [1] |
| rs28394864 | 17 | 47450775 | ABI3 | A/G | 0.473 | Yes | [1] |
| rs3752246 | 19 | 1056492 | ABCA7 | G/C | 0.182 | Yes | [2] |
| rs3865444 | 19 | 51727962 | CD33 | A/C | 0.32 | Yes | [1] |
| rs6024870 | 20 | 54997568 | CASS4 | A/G | 0.088 | Yes | [2] |
| rs429358 | 19 | 4908684 | APOE | T/C | 0.14 | Yes |  |
| rs2830500 | 21 | 28156856 | ADAMTS1 | A/C | 0.308 | Yes | [2] |
| rs7657553 | 3 | 57226150 | HESX1 | A/G | 0.205 | No | [1] |
| rs75932628 | 6 | 41129252 | TREM2 | T/C | 0.008 | No | [2] |
| rs9473117 | 6 | 47431284 | CD2AP | C/A | 0.28 | No | [2] |
| rs114360492 | 7 | 145950029 | CNTNAP2 | T/C | 2.59*10-4 | No | [1] |
| rs11218343 | 11 | 121435587 | SORL1 | C/T | 0.04 | No | [2] |
| rs117618017 | 15 | 63569902 | APH1B | T/C | 0.132 | No | [1] |
| rs62039712 | 16 | 79355857 | WWOX/MAF | A/G | 0.116 | No | [2] |
| rs138190086 | 17 | 61538148 | ACE | A/G | 0.02 | No | [2] |
| rs76726049 | 18 | 56189459 | ALPK2 | C/T | 0.014 | No | [1] |
| rs76320948 | 19 | 46241841 | BHMG1 | T/C | 0.046 | No | [1] |

Genetic variants associated with AD at p<5e-8 with AD in Genome Wide Association and meta-analyses (GWAMA) studies published until December 2019 were used. Variants from Kunkle *et al.* [2]were primarily used as this was the largest GWAMA with clinical AD diagnosis. Additional variants that were not identified by Kunkle *et al.* but reached genome-wide significance in five large GWA and GWAMA studies [1], [3]–[5] were also included. For the APOE gene, rs429358 was included; additionally the APOE4 genotype was genotyped as part of this study (using TAQMAN) and was investigated for associations with the modules. Variants not include in our study were not available in our dataset, usually due to low minor allele frequency.

*rs35103166 (R^2=0.50); **rs7570061 (R^2=0.75); ***rs4236673 (R^2=0.9).

**Table S2. Top 10 drivers in five selected lipid modules.**

| Module | Lipid feature/Putative annotation | kME |
| --- | --- | --- |
| darkturquoise | 604.54_29.19 | 0.93 |
|  | 603.54_29.19 | 0.93 |
|  | 578.53_29.19 | 0.91 |
|  | 577.52_29.19 | 0.90 |
|  | 859.78_29.17 | 0.88 |
|  | 638.57_19.38 / DG (36:2) | 0.88 |
|  | 890.45_29.19 | 0.87 |
|  | 579.54_29.19 | 0.87 |
|  | 878.82_29.2 / TG (52:1) | 0.85 |
|  | 639.58_19.4 | 0.85 |
| green | 688.6_24.91 / ChE (20:5) | 0.85 |
|  | 971.8_26.63 | 0.85 |
|  | 689.61_24.91 | 0.84 |
|  | 949.81_27.97 | 0.85 |
|  | 303.24_24.9 | 0.85 |
|  | 970.79_26.62 / TG (60:11) | 0.84 |
|  | 966.76_25.04 | 0.84 |
|  | 672.58_24.9 | 0.84 |
|  | 969.78_25.85 | 0.84 |
|  | 690.61_24.91 | 0.83 |
| midnightblue | 398.4_27.8 | 0.91 |
|  | 397.39_27.8 | 0.91 |
|  | 398.4_29.27 | 0.90 |
|  | 397.39_29.28 | 0.90 |
|  | 681.64_26.99 / ChE (20:0) | 0.89 |
|  | 680.64_27.01 | 0.89 |
|  | 682.65_26.98 | 0.89 |
|  | 384.38_27 | 0.88 |
|  | 399.4_27.8 | 0.87 |
|  | 383.37_27.01 | 0.87 |
| greenyellow | 670.65_28.82 | 0.76 |
|  | 370.36_28.83 | 0.75 |
|  | 632.64_21.49 | 0.75 |
|  | 671.66_28.82 | 0.74 |
|  | 369.36_28.96 | 0.74 |
|  | 619.63_20.87 | 0.73 |
|  | 633.64_21.48 | 0.72 |
|  | 618.62_20.87 | 0.72 |
|  | 651.65_21.51 | 0.71 |
|  | 636.63_20.87 / Cer(d41:1) | 0.70 |
| orange | 795.32_18.56 | 0.72 |
|  | 773.31_16.99 | 0.70 |
|  | 794.94_18.55 | 0.68 |
|  | 183.51_16.97 | 0.68 |
|  | 762.06_16.99 | 0.68 |
|  | 761.04_16.98 | 0.63 |
|  | 630.62_20.45 | 0.58 |
|  | 631.62_20.46 | 0.57 |
|  | 791.63_18.57 | 0.57 |
|  | 634.65_21.53 / Cer(d42:0) | 0.57 |

kME represents the module membership value for individual protein in assigned module.

**Table S3. Selected lipid modules and annotation.**

| Module | Association with phenotypes | Lipid species |
| --- | --- | --- |
| green | Case-control, Hip (L) | PE, PC, TG |
| darkturquoise | ROD, Hip(L) | DG, TG |
| midnightblue | ROD, Hip(L), Hip(R) | ChE, TG |
| greenyellow | Hip(R), ERC(L) | SMs, PCs, Cers, TGs |
| orange | Hip(L), Hip(R), ERC(L), ERC(R) | PC, DG |

PE, phosphatidylethanolamine; PC, phosphatidylcholine; TG, triglyceride; DG, diacylglyceride; CE, cholesteryl ester; SM, sphingomyelin; Cer, Ceramide.

**Table S4. Top 10 drivers in five selected protein modules.**

| Module | Uniprot ID | Abbreviations | Protein | kME |
| --- | --- | --- | --- | --- |
| lightgreen | P05186 | Alkaline phosphatase, bone | Alkaline phosphatase | 0.81 |
|  | P17213 | BPI | Bactericidal permeability-increasing protein | 0.79 |
|  | P02788 | Lactoferrin | Lactotransferrin | 0.76 |
|  | P78380 | LOX-1 | Oxidized low-density lipoprotein receptor 1 | 0.70 |
|  | P14780 | MMP-9 | Matrix metalloproteinase-9 | 0.69 |
|  | P16403 | Histone H1.2 | Histone H1.2 | 0.65 |
|  | O75594 | PGRP-S | Peptidoglycan recognition protein 1 | 0.50 |
|  | P07451 | Carbonic.anhydrase.III | Carbonic anhydrase 3 | 0.29 |
|  | P02649 | apoE4 | Apolipoprotein E4 | 0.22 |
|  | P02649 | apoE3 | Apolipoprotein E3 | 0.20 |
| lightcyan | Q96KQ7 | HMTase G9a | Histone-lysine N-methyltransferase EHMT2 | 0.65 |
|  | P01282 | Vasoactive Intestinal Peptide | Vasoactive.Intestinal.Peptides | 0.61 |
|  | P20783 | Neurotrophin-3 | Neurotrophin-3 | 0.58 |
|  | P00734 | Thrombin | Prothrombin | 0.56 |
|  | P01031 | C5 | Complement C5 | 0.53 |
|  | Q96P31 | FCRL3 | Fc receptor-like protein 3 | 0.53 |
|  | P22079 | Lactoperoxidase | Lactoperoxidase | 0.52 |
|  | P01031 | C5b,6 Complex | Complement C5b-C6 | 0.50 |
|  | P22223 | P-Cadherin | Cadherin-3 | 0.50 |
|  | P16860 | BNP-32 | Natriuretic peptides B | 0.50 |
| cyan | O95243 | MBD4 | Methyl-CpG-binding domain protein 4 | 0.66 |
|  | Q969D9 | TSLP | Thymic stromal lymphopoietin | 0.56 |
|  | Q9UHX3 | EMR2 | Adhesion G protein-coupled receptor E2 | 0.52 |
|  | P43166 | Carbonic.anhydrase.VII | Carbonic anhydrase 7 | 0.52 |
|  | P08311 | Cathepsin.G | Cathepsin G | 0.51 |
|  | P03372 | ER | Estrogen receptor | 0.51 |
|  | Q9HC73 | TSLP R | Cytokine receptor-like factor 2 | 0.49 |
|  | P09758 | GA733-1 protein | Tumor-associated calcium signal transducer 2 | 0.46 |
|  | P28908 | CD30 | Tumor necrosis factor receptor superfamily member 8 | 0.41 |
|  | Q9H293 | IL-17E | Interleukin-25 | 0.31 |
| red | P19438 | TNF sR-I | Tumor necrosis factor receptor superfamily member 1A | 0.74 |
|  | Q6NW40 | RGM-B | RGM domain family member B | 0.73 |
|  | P00746 | Factor D | Complement factor D | 0.69 |
|  | P03973 | SLPI | Antileukoproteinase | 0.64 |
|  | Q16627 | HCC-1 | C-C motif chemokine 14 | 0.60 |
|  | P61626 | Lysozyme | Lysozyme C | 0.58 |
|  | P80188 | Lipocalin 2 | Neutrophil gelatinase-associated lipocalin | 0.58 |
|  | Q03405 | suPAR | Urokinase plasminogen activator surface receptor | 0.58 |
|  | P30040 | ERp29 | Endoplasmic reticulum resident protein 29 | 0.57 |
|  | Q16663 | MIP-5 | C-C motif chemokine 15 | 0.56 |
| yellow | Q13478 | IL-18 Ra | Interleukin-18 receptor 1 | 0.64 |
|  | Q9Y286 | Siglec-7 | Sialic acid-binding Ig-like lectin 7 | 0.55 |
|  | P21810 | Biglycan | Biglycan | 0.53 |
|  | O60486 | Plexin C1 | Plexin-C1 | 0.48 |
|  | Q03154 | Aminoacylase-1 | Aminoacylase-1 | 0.47 |
|  | P05156 | Factor I | Complement factor I | 0.46 |
|  | P00797 | Renin | Renin | 0.44 |
|  | P01833 | pIgR | Polymeric immunoglobulin receptor | 0.43 |
|  | Q6UWV6 | ENPP7 | Ectonucleotide pyrophosphatase/phosphodiesterase family member 7 | 0.42 |
|  | P12830 | Cadherin-1 | Cadherin-1 | 0.41 |

kME represents the module membership value for individual protein in assigned module.

**Table S5. Summary of gene set enrichment analyses and gene ontology enrichment analysis of protein modules.**

| **Modules** | **Biological process** | **Cellular component** | **Molecular function** | **KEGG pathway** | **Reactome pathway** |
| --- | --- | --- | --- | --- | --- |
| **lightgreen** | Neutrophil mediated immunity | Vesicle lumen | Peptidase regulator activity | Cytokine-cytokine receptor interaction | Neutrophil degranulation |
|  | Granulocyte activation | Specific granule | Glycosaminoglycan binding | Osteoclast differentiation | Antimicrobial peptides |
|  | Multi-organism cellular process | Tertiary granule | Immunoglobulin binding | Phagosome | Innate immune system |
|  | STAT cascade | Blood microparticle | Lipopolysaccharide binding | JAK-STAT signaling pathway | Immune System |
|  | Negative regulation of response to external stimulus | Extracellular matrix | Proteoglycan binding | Thiamine metabolism | Binding and uptake of ligands by scavenger receptors |
|  | Biomineral tissue development | Endocytic vesicle | Enzyme inhibitor activity | Nitrogen metabolism | Chylomicron clearance |
|  | Modification of morphology or physiology of other organism | Receptor complex | Sulfur compound binding | Transcriptional misregulation in cancer | Post-translational modification: synthesis of gpi-anchored proteins |
|  | Cell killing | Platelet alpha granule | Transmembrane receptor protein kinase activity | Pathways in cancer | Metal sequestration by antimicrobial proteins |
|  | Negative regulation of proteolysis | Anchored component of membrane | Cytokine binding | Folate biosynthesis | Post-translational protein phosphorylation |
|  | Regulation of inflammatory response | Endolysosome | Protein tyrosine kinase activity | Thyroid cancer | Chylomicron remodeling |
|  | Ossification |  |  |  |  |
|  | Peptidyl-tyrosine modification |  |  |  |  |
|  | Regulation of peptidase activity |  |  |  |  |
|  | Negative regulation of hydrolase activity |  |  |  |  |
|  | Humoral immune response |  |  |  |  |
|  | Leukocyte differentiation |  |  |  |  |
|  | Regulation of protein serine/threonine kinase activity |  |  |  |  |
|  | Tumor necrosis factor superfamily cytokine production |  |  |  |  |
|  | Tissue remodeling |  |  |  |  |
|  | Regulation of innate immune response |  |  |  |  |
|  | Ameboidal-type cell migration |  |  |  |  |
|  | Cytolysis |  |  |  |  |
|  | Negative regulation of cell activation |  |  |  |  |
|  | Leukocyte migration |  |  |  |  |
| **cyan** | Positive regulation of cytokine production | Vesicle lumen | Receptor ligand activity | Cytokine-cytokine receptor interaction | Immune system |
|  | Mast cell activation | Platelet alpha granule | Serine hydrolase activity | JAK-STAT signaling pathway | Signaling by interleukins |
|  | Tumor necrosis factor superfamily cytokine production | Specific granule | Endopeptidase activity | Complement and coagulation cascades | Cytokine signaling in immune system |
|  | Interleukin-5 production | Receptor complex | Cytokine binding | Pathways in cancer | Interleukin-4 and interleukin-13 signaling |
|  | Protein maturation | Side of membrane | Cytokine receptor binding | Adipocytokine signaling pathway | Terminal pathway of complement |
|  | Leukocyte activation involved in inflammatory response | Extracellular matrix | Growth factor receptor binding | Il-17 signaling pathway | Regulation of complement cascade |
|  | Regulation of inflammatory response | Blood microparticle | Phosphatidylinositol bisphosphate kinase activity | Amoebiasis | Complement cascade |
|  | Regulation of immune effector process | Apical part of cell | Phosphatidylinositol 3-kinase activity | Hematopoietic cell lineage | Innate immune system |
|  | Peptidyl-tyrosine modification | Tertiary granule | Cytokine receptor activity | Endocrine resistance | Activation of matrix metalloproteinases |
|  | Humoral immune response | Perinuclear endoplasmic reticulum | Guanyl-nucleotide exchange factor activity | Nitrogen metabolism | Mapk1/mapk3 signaling |
|  | Interleukin-6 production |  |  |  |  |
|  | Neuroinflammatory response |  |  |  |  |
|  | STAT cascade |  |  |  |  |
|  | Acute inflammatory response |  |  |  |  |
|  | Regulation of leukocyte activation |  |  |  |  |
|  | Positive regulation of cell activation |  |  |  |  |
|  | Reactive nitrogen species metabolic process |  |  |  |  |
|  | Macrophage activation |  |  |  |  |
|  | Cytokine secretion |  |  |  |  |
|  | Protein activation cascade |  |  |  |  |
|  | Protein kinase B signaling |  |  |  |  |
|  | Interleukin-13 production |  |  |  |  |
|  | Reactive oxygen species metabolic process |  |  |  |  |
| **lightcyan** | Protein activation cascade | Endoplasmic reticulum lumen | Receptor ligand activity | Complement and coagulation cascades | Transport of gamma-carboxylated protein precursors from the endoplasmic reticulum to the golgi apparatus |
|  | Insulin-like growth factor receptor signaling pathway | Golgi lumen | Cytokine receptor binding | P53 signaling pathway | Gamma-carboxylation of protein precursors |
|  | Epithelial cell proliferation | Protein-lipid complex | Phospholipid binding | Longevity regulating pathway | Removal of aminoterminal propeptides from gamma-carboxylated proteins |
|  | Regulation of chemotaxis | Platelet alpha granule | Insulin receptor binding | Vitamin digestion and absorption | Gamma-carboxylation, transport, and amino-terminal cleavage of proteins |
|  | Leukocyte migration | Blood microparticle | Hormone receptor binding | Glycosphingolipid biosynthesis | Regulation of insulin-like growth factor (igf) transport and uptake by insulin-like growth factor binding proteins (igfbps) |
|  | Regulation of plasma lipoprotein particle levels | Vesicle lumen | Cell adhesion molecule binding | Transcriptional misregulation in cancer | Formation of fibrin clot (clotting cascade) |
|  | Cell growth | Endoplasmic reticulum exit site | Fibronectin binding | Prion diseases | Gamma carboxylation, hypusine formation and arylsulfatase activation |
|  | Regulation of body fluid levels | Smooth endoplasmic reticulum | Lipoprotein particle receptor binding | Aldosterone-regulated sodium reabsorption | Regulation of complement cascade |
|  | Positive regulation of response to external stimulus | Endosome lumen | Serine hydrolase activity | Fat digestion and absorption | Complement cascade |
|  | Protein-containing complex remodeling | Extrinsic component of membrane | Peptidase regulator activity | Ras signaling pathway | Synthesis, secretion, and deacylation of ghrelin |
| **red** | Humoral immune response | Vesicle lumen | Receptor ligand activity | Cytokine-cytokine receptor interaction | Immune system |
|  | Response to tumor necrosis factor | Extracellular matrix | Serine hydrolase activity | Mapk signaling pathway | Regulation of insulin-like growth factor (igf) transport and uptake by insulin-like growth factor binding proteins (igfbps) |
|  | Leukocyte migration | Specific granule | Endopeptidase activity | Rap1 signaling pathway | Signaling by interleukins |
|  | Cell chemotaxis | Endoplasmic reticulum lumen | Protein tyrosine kinase activity | Fluid shear stress and atherosclerosis | Cytokine signaling in immune system |
|  | Response to interleukin-1 | Vacuolar lumen | Phosphatidylinositol bisphosphate kinase activity | Nf-kappa b signaling pathway | Neutrophil degranulation |
|  | Neutrophil mediated immunity | Primary lysosome | Glycosaminoglycan binding | Hif-1 signaling pathway | Activation of matrix metalloproteinases |
|  | Granulocyte activation | Anchored component of membrane | Phosphatidylinositol 3-kinase activity | Tuberculosis | Innate immune system |
|  | Positive chemotaxis | Extrinsic component of membrane | Enzyme inhibitor activity | Kaposi sarcoma-associated herpesvirus infection | Degradation of the extracellular matrix |
|  | Positive regulation of response to external stimulus | Ficolin-1-rich granule | Cytokine receptor binding | Chemokine signaling pathway | Interleukin-10 signaling |
|  | Regulation of vasculature development | Pigment granule | Peptidase regulator activity | Osteoclast differentiation | Post-translational protein phosphorylation |
|  | Negative regulation of hydrolase activity |  |  |  |  |
|  | Negative regulation of proteolysis |  |  |  |  |
|  | Defense response to other organism |  |  |  |  |
|  | Angiogenesis |  |  |  |  |
|  | Adaptive immune response |  |  |  |  |
|  | Peptidyl-tyrosine modification |  |  |  |  |
|  | Extracellular structure organization |  |  |  |  |
|  | ERK1 and ERK2 cascade |  |  |  |  |
|  | Production of molecular mediator of immune response |  |  |  |  |
|  | Response to interferon-gamma |  |  |  |  |
|  | Regulation of peptidase activity |  |  |  |  |
|  | Response to mechanical stimulus |  |  |  |  |
|  | Platelet degranulation |  |  |  |  |
|  | Multi-multicellular organism process |  |  |  |  |
|  | Transmembrane receptor protein serine/threonine kinase signaling pathway |  |  |  |  |
|  | T cell activation |  |  |  |  |
|  | Response to oxygen levels |  |  |  |  |
| **yellow** | Humoral immune response | Extracellular matrix | Serine hydrolase activity | Staphylococcus aureus infection | Platelet degranulation |
|  | Protein activation cascade | Vesicle lumen | Endopeptidase activity | Cytokine-cytokine receptor interaction | Response to elevated platelet cytosolic ca2+ |
|  | Platelet degranulation | Blood microparticle | Receptor ligand activity | Phagosome | Immune system |
|  | Adaptive immune response | Platelet alpha granule | Immunoglobulin binding | Complement and coagulation cascades | Extracellular matrix organization |
|  | Extracellular structure organization | Vacuolar lumen | Carbohydrate binding | Tuberculosis | Complement cascade |
|  | ERK1 and ERK2 cascade | Side of membrane | Proteoglycan binding | Malaria | Innate Immune System |
|  | Acute inflammatory response | Receptor complex | Transmembrane receptor protein kinase activity | Tgf-beta signaling pathway | Initial triggering of complement |
|  | Regulation of immune effector process | Endoplasmic reticulum lumen | Cytokine receptor binding | Intestinal immune network for iga production | Platelet activation, signaling and aggregation |
|  | Negative regulation of cell adhesion | Endolysosome | Protein tyrosine kinase activity | Leishmaniasis | Formation of fibrin clot (clotting cascade) |
|  | Granulocyte activation | Primary lysosome | Growth factor binding | Egfr tyrosine kinase inhibitor resistance | Neutrophil degranulation |
|  | Regulation of inflammatory response |  | Peptidase regulator activity |  |  |
|  | Lymphocyte mediated immunity |  | Cytokine receptor activity |  |  |
|  | Neutrophil mediated immunity |  | Extracellular matrix structural constituent |  |  |
|  | Negative regulation of proteolysis |  |  |  |  |
|  | Collagen metabolic process |  |  |  |  |
|  | Regulation of vesicle-mediated transport |  |  |  |  |
|  | Leukocyte cell-cell adhesion |  |  |  |  |
|  | Protein maturation |  |  |  |  |
|  | Regulation of peptidase activity |  |  |  |  |
|  | Negative regulation of hydrolase activity |  |  |  |  |
|  | Connective tissue development |  |  |  |  |
|  | Defense response to other organism |  |  |  |  |
|  | Receptor-mediated endocytosis |  |  |  |  |
|  | Regulation of cell-cell adhesion |  |  |  |  |
|  | Cell recognition |  |  |  |  |
|  | Response to interleukin-1 |  |  |  |  |
|  | Modification of morphology or physiology of other organism |  |  |  |  |
|  | Positive regulation of defense response |  |  |  |  |
|  | Cell killing |  |  |  |  |
|  | Phagocytosis |  |  |  |  |
|  | Cell-substrate adhesion |  |  |  |  |
|  | Gland development |  |  |  |  |
|  | Response to tumor necrosis factor |  |  |  |  |
|  | Epithelial cell apoptotic process |  |  |  |  |
|  | Regulation of peptide secretion |  |  |  |  |
|  | Mesenchyme development |  |  |  |  |
|  | Response to fungus |  |  |  |  |
|  | Interleukin-10 production |  |  |  |  |
|  | Protein-lipid complex subunit organization |  |  |  |  |
|  | Exocrine system development |  |  |  |  |
|  | Response to nutrient levels |  |  |  |  |
|  | Production of molecular mediator of immune response |  |  |  |  |
|  | Response to interferon-gamma |  |  |  |  |
|  | Peptidyl-tyrosine modification |  |  |  |  |
|  | Positive regulation of cell adhesion |  |  |  |  |
|  | Heterotypic cell-cell adhesion |  |  |  |  |
|  | Positive regulation of secretion |  |  |  |  |
|  | Odontogenesis |  |  |  |  |

GO terms and pathways do not pass Benjamini-Hochberg FDR correction were highlighted in tan.

**Table S6. Correlations between lipid modules/protein modules and AD genetic variants.**

| Lipid modules | Locus | Variant | chromosome | *p* value |
| --- | --- | --- | --- | --- |
| green | MEF2C | rs190982 | 5 | 4.75e-03 |
|  | ABCA7 | rs3752246 | 19 | 3.80e-02 |
| darkturquoise | IL-34 | rs4985556 | 16 | 1.20e-03 |
|  | MEF2C | rs190982 | 5 | 5.50e-03 |
|  | CR1 | rs4844610 | 1 | 1.23e-02 |
|  | ANKRD31 | RS4704171 | 5 | 2.78e-02 |
| Midnightblue | FERMT2  PLCG2 | rs17125924  rs12444183 | 14  16 | 4.69e-02  2.03e-02 |
| greenyellow | ANKRD31 | Rs4704171 | 5 | 3.90e-02 |
| Protein modules | **Locus** | **Variant** | **chromosome** | ***p* value** |
| lightgreen | IQCK | rs7185636 | 16 | 2.48e-02 |
|  | PTK2B  HLA-DRB1 | rs73223431  rs9271058 | 8  6 | 2.50e-02  2.92e-02 |
| red | MS4A6A | rs7933202 | 11 | 7.90e-03 |
|  | HLA -DRB1 | rs9271058 | 6 | 3.30e-02 |
| lightcyan | CASS4 | rs6024870 | 20 | 2.60e-03 |
|  | APOE | rs429358  APOE4 genotype* | 19 | 1.25e-02  3.0e-03 |
|  | HLA -DRB1 | rs9271058 | 6 | 1.64e-02 |
| cyan | NDUFAF6 | rs4735340 | 8 | 4.69e-03 |
|  | IL-34 | rs4985556 | 16 | 1.35e-02 |
|  | PICALM | rs3851179 | 11 | 4.01e-02 |
|  | APOE | rs429358  APOE4 genotype* | 19 | 4.8e-02  2.0e-02 |

* The APOE4 genotype was genotyped using TAQMAN


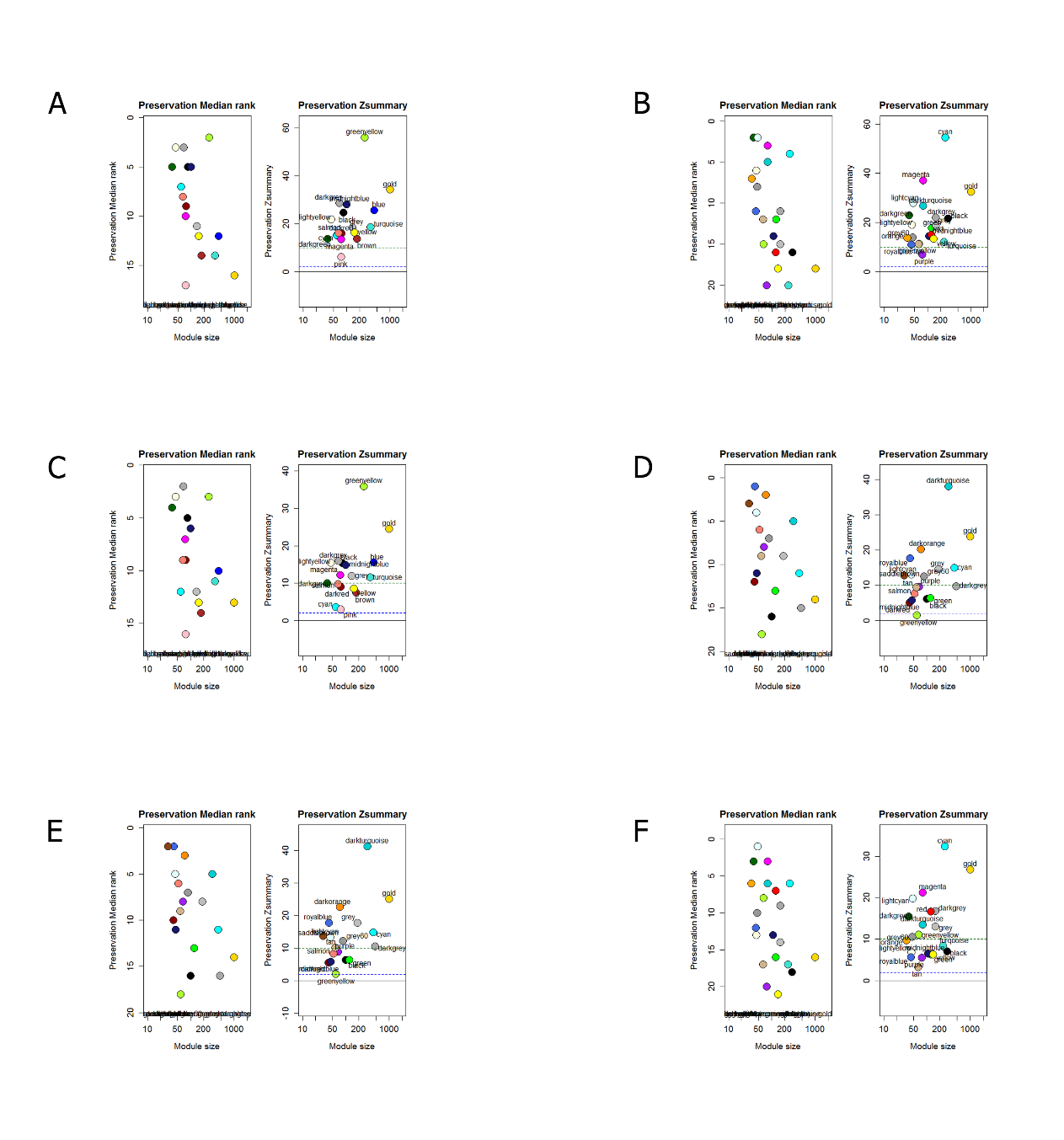


**Figure S1. Preservation summary plots for AD, MCI and control sub datasets in lipidomics dataset.** A. AD sub dataset (test) and control sub dataset (reference); B. control sub dataset (test) and AD sub dataset (reference); C. AD sub dataset (test) and MCI sub dataset (reference); D. MCI sub dataset (test) and AD sub dataset (reference); E. MCI sub dataset (test) and control sub dataset (reference); F. control sub dataset (test) and MCI sub dataset (reference). Z*_summary_* is the summary preservation statistics. Y-axis represents preservation statistics for the corresponding module in the case data sets, and x-axis is the lipid feature numbers in each module. The dashed blue and green lines indicate the thresholds Z=2 and Z=10, respectively. Z*_summary_* < 2 implies no evidence for module preservation, 2 < Z*_summary_* < 10 implies weak to moderate evidence, and Z*_summary_* > 10 implies strong evidence for module preservation.


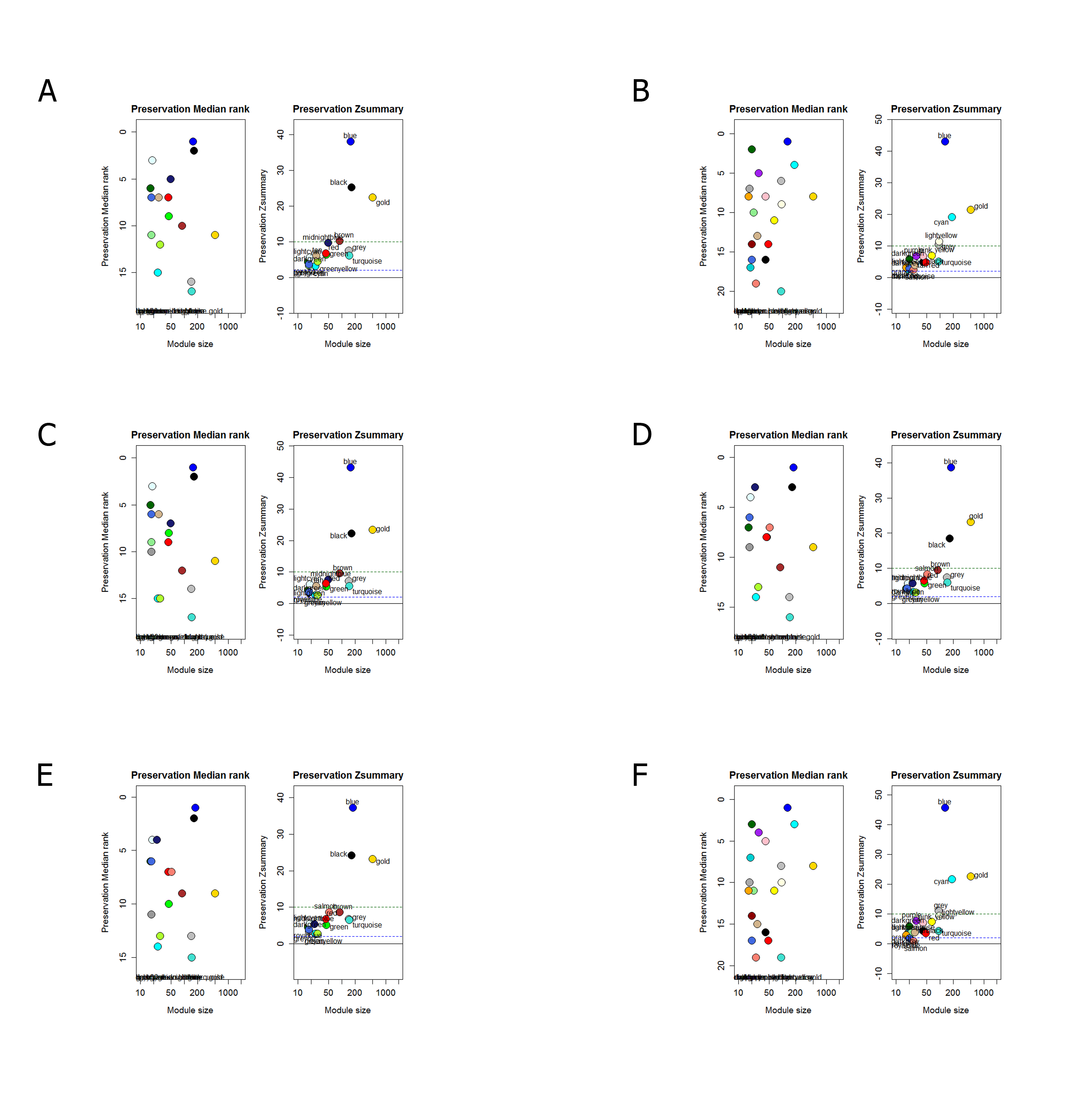


**Figure S2.** **Preservation summary plots for AD, MCI and control sub datasets in ANM proteomics dataset.** A. AD sub dataset (test) and control sub dataset (reference); B. control sub dataset (test) and AD sub dataset (reference); C. AD sub dataset (test) and MCI sub dataset (reference); D. MCI sub dataset (test) and AD sub dataset (reference); E. MCI sub dataset (test) and control sub dataset (reference); F. control sub dataset (test) and MCI sub dataset (reference). Z*_summary_* is the summary preservation statistics. Y-axis represents preservation statistics for the corresponding module in the case data sets, and x-axis is the lipid feature numbers in each module. The dashed blue and green lines indicate the thresholds Z=2 and Z=10, respectively. Z*_summary_* < 2 implies no evidence for module preservation, 2 < Z*_summary_* < 10 implies weak to moderate evidence, and Z*_summary_* > 10 implies strong evidence for module preservation.


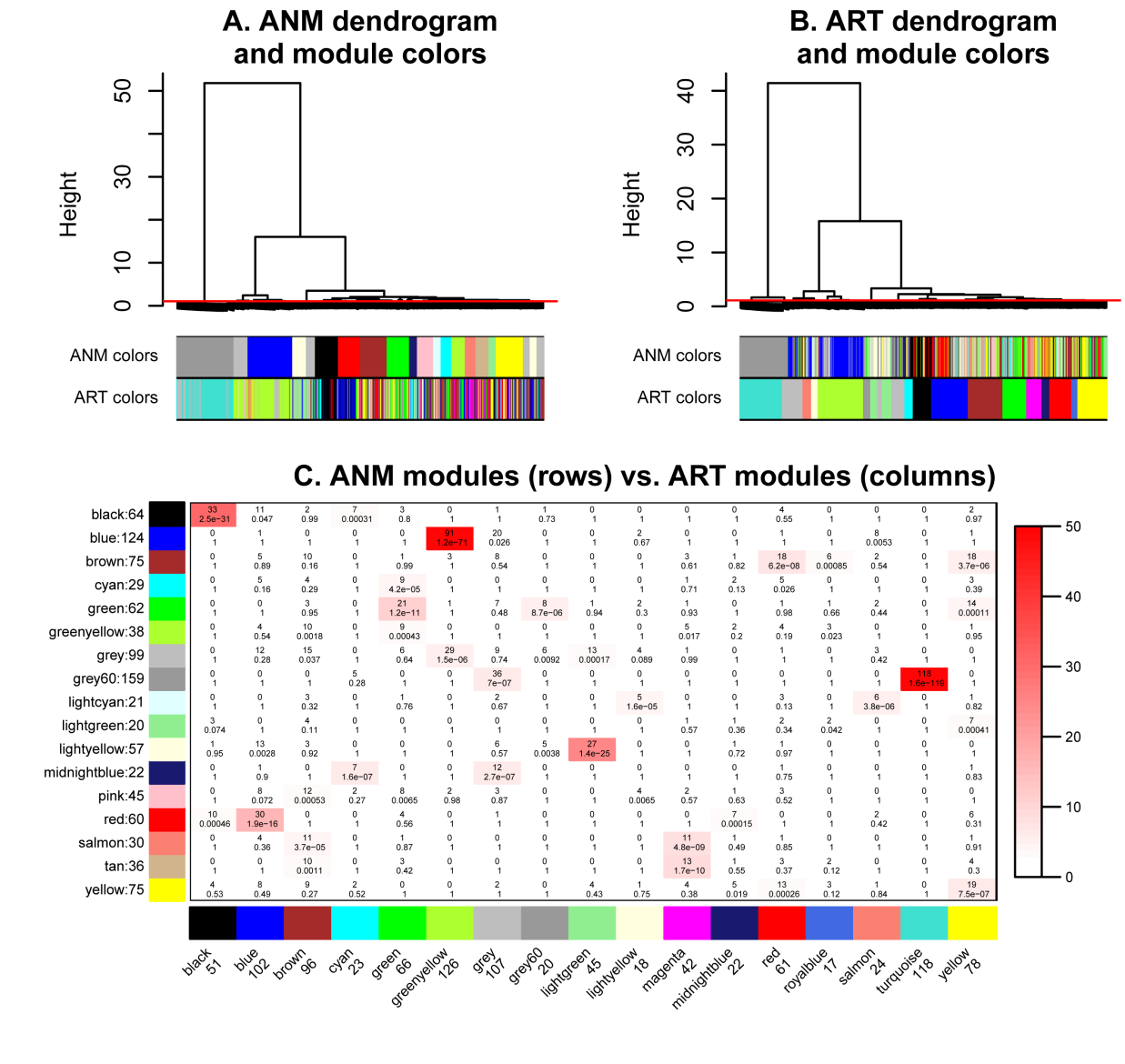


**Figure S3. Dendrogram and cross-tabulation based comparison of modules in ANM and ART protein cohort networks.** A. Hierarchical clustering tree (dendrogram) of proteins based on ANM cohort network. The color rows below the dendrogram indicate module membership in the ANM modules (defined by cutting branches of this dendrogram at the red line) and in the ART network (defined by branch cutting the dendrogram in panel B.) B. Hierarchical clustering tree of proteins based on the ART co-expression network. The color rows below the dendrogram indicate module membership in the human modules (defined by cutting branches of dendrogram in panel A.) and in the ART network (defined by branch cutting the dendrogram in this panel.) C. Cross-tabulation of ANM modules (rows) and ART modules (columns). Each row and column is labeled by the corresponding module color and the total number of proteins in the module. In the table, numbers give counts of proteins in the intersection of the corresponding row and column module. The table is color-coded by -log(p), the Fisher exact test p value, according to the color legend on the right.


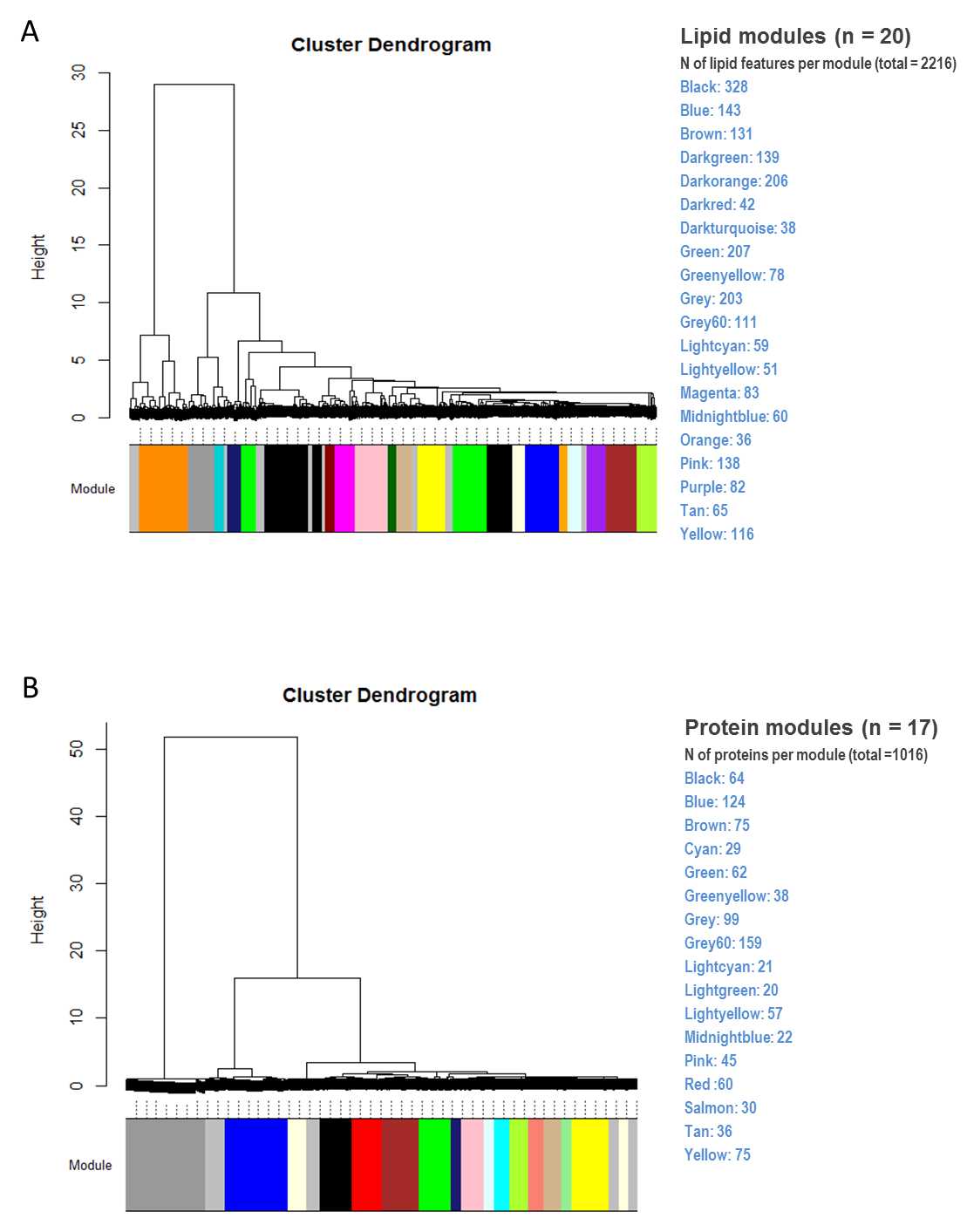


**Figure S4.** A. Cluster dendrogram of weighted lipid correlation network analysis and the number of lipid features in each module; B. Cluster dendrogram of weighted protein correlation network analysis and the number of proteins in each module.


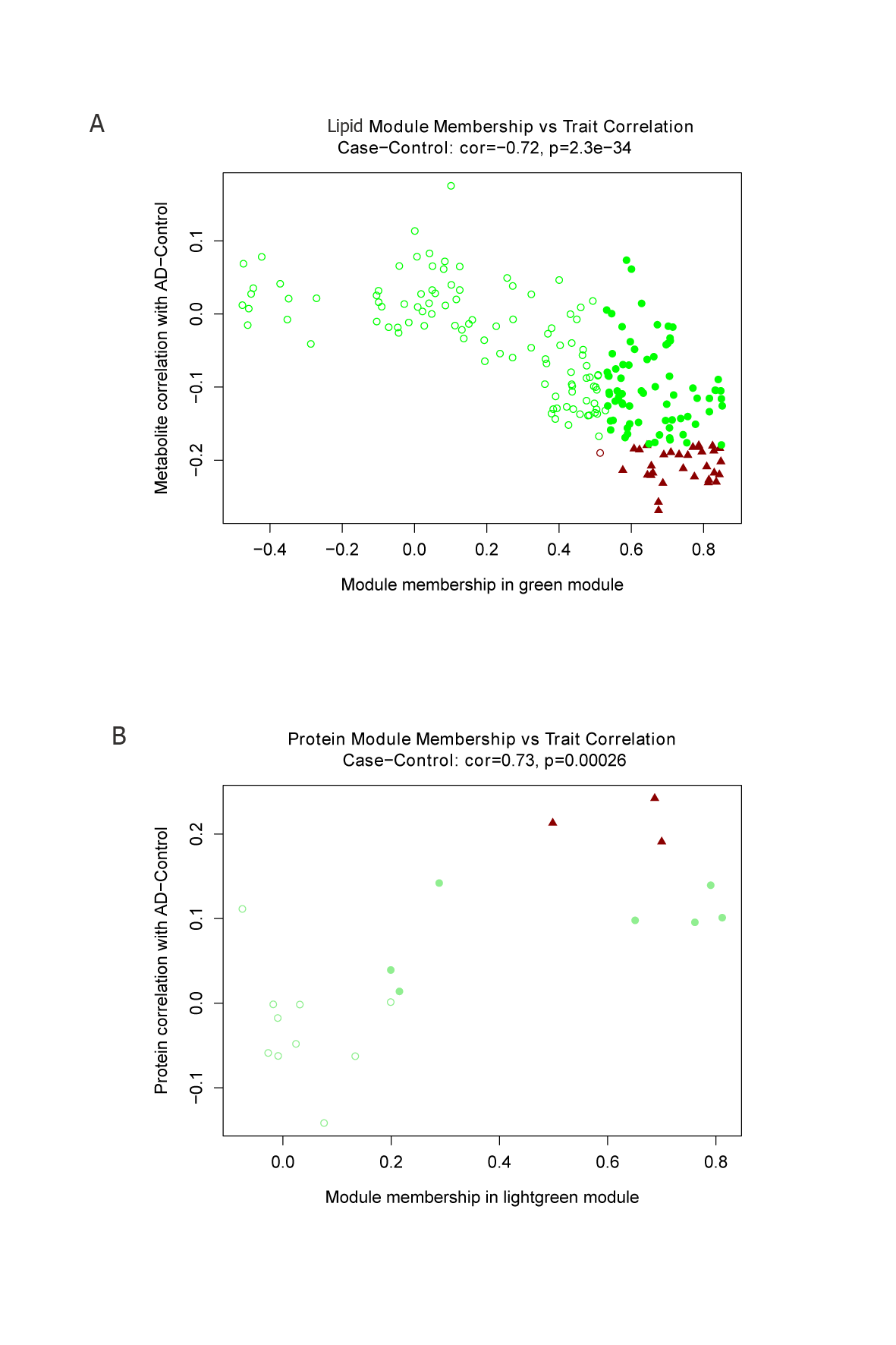


**Figure S5.** **Scatter plots of module membership versus lipids/proteins-diagnosis correlation.** (A) Lipid module membership for green module vs lipid-diagnosis correlation; (B) Protein module membership for lightgreen module vs protein-diagnosis correlation. Filled circles in represent lipids/proteins with MM > median & correlation q value (Bonferroni correction) > 0.05; filled dark red triangles represents lipid/proteins with MM > median & correlation q value (Bonferroni correction) < 0.05; hollow dark red circles represent lipid/proteins with MM < median & correlation q value (Bonferroni correction) < 0.05.


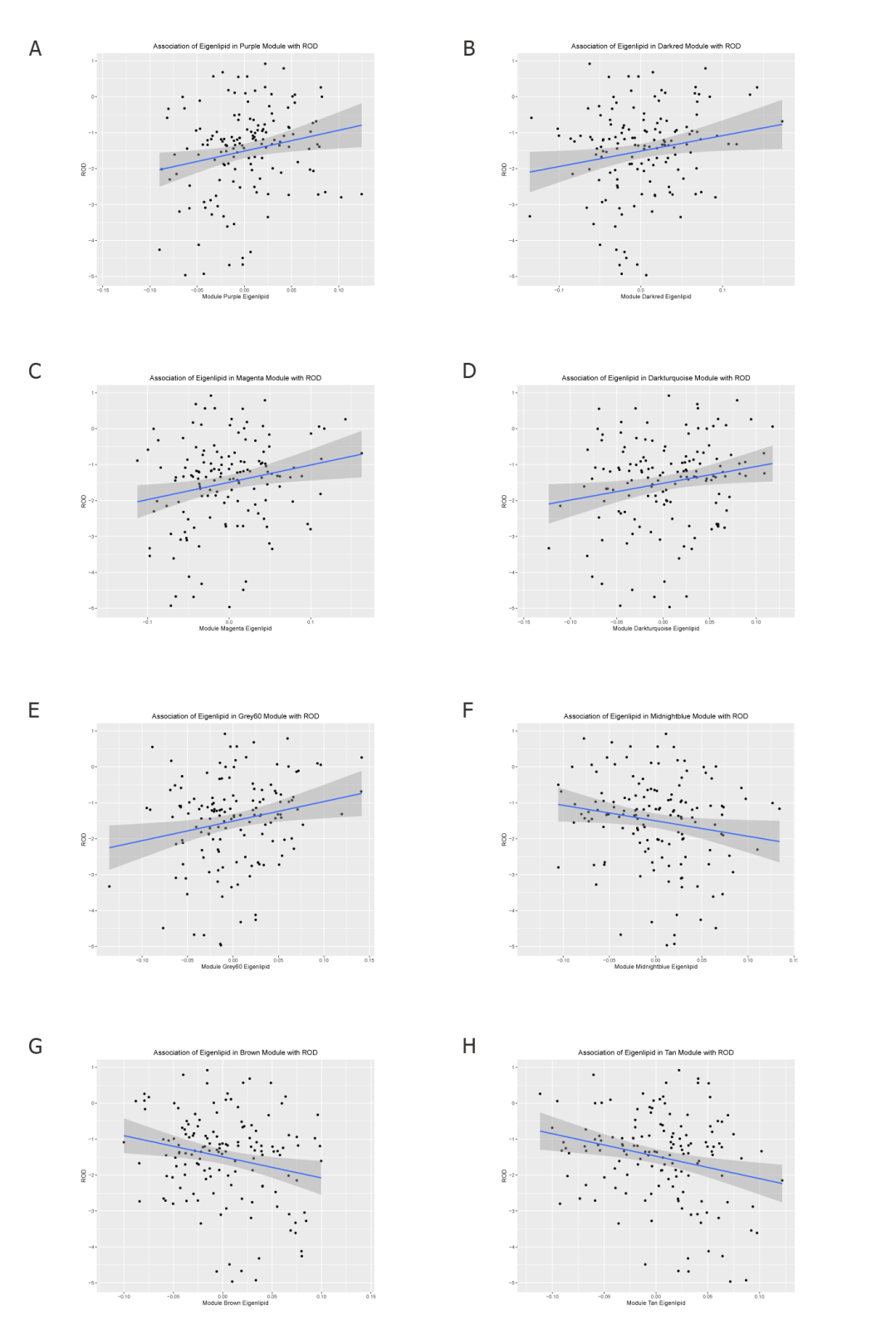


**Figure S6.** **Scatter plots of eigenlipids correlation with ROD.** (A) Eigenlipid in purple module; (B) Eigenlipid in darkred module; (C) Eigenlipid in magenta module; (D) Eigenlipid in darkturquoise module; (E) Eigenlipid in grey60 module; (F) Eigenlipid in midnightblue module; (G) Eigenlipid in brown module; (H) Eigenlipid in tan module.


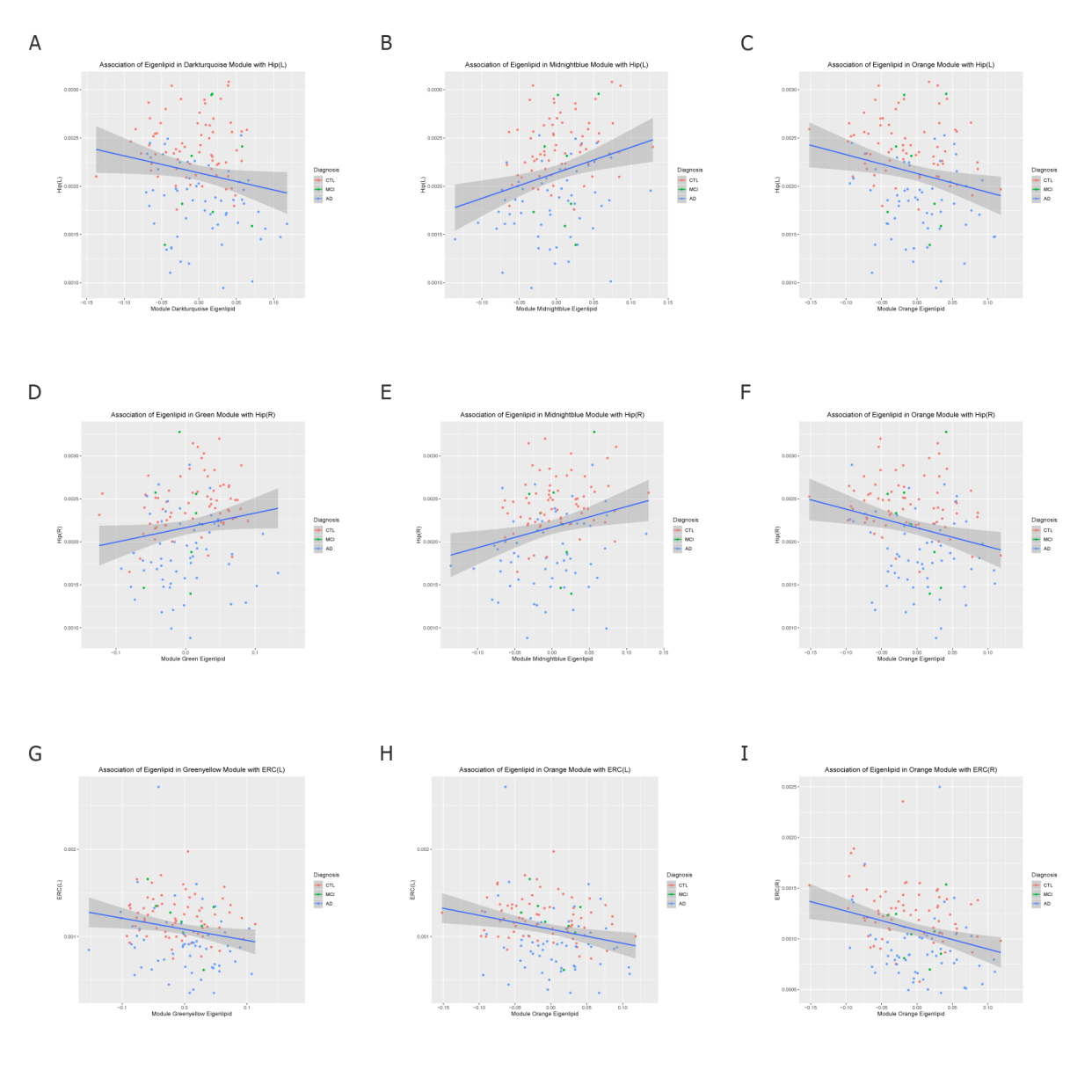


**Figure S7.** **Scatter plots of eigenlipids correlation with brain atrophy measures.** (A) Eigenlipid in darkturquoise module vs. Hippocampal left volume ; (B) Eigenlipid in midnightblue module vs. Hippocampal left volume; (C) Eigenlipid in orange module vs. Hippocampal left volume; (D) Eigenlipid in green module vs. Hippocampal right volume; (E) Eigenlipid in midnightblue module vs. Hippocampal right volume; (F) Eigenlipid in orange module vs. Hippocampal right volume; (G) Eigenlipid in greenyellow module vs. entorhinal cortex left volume; (H) Eigenlipid in orange module vs. entorhinal cortex left volume; (I) Eigenlipid in orange module vs. entorhinal cortex right volume.


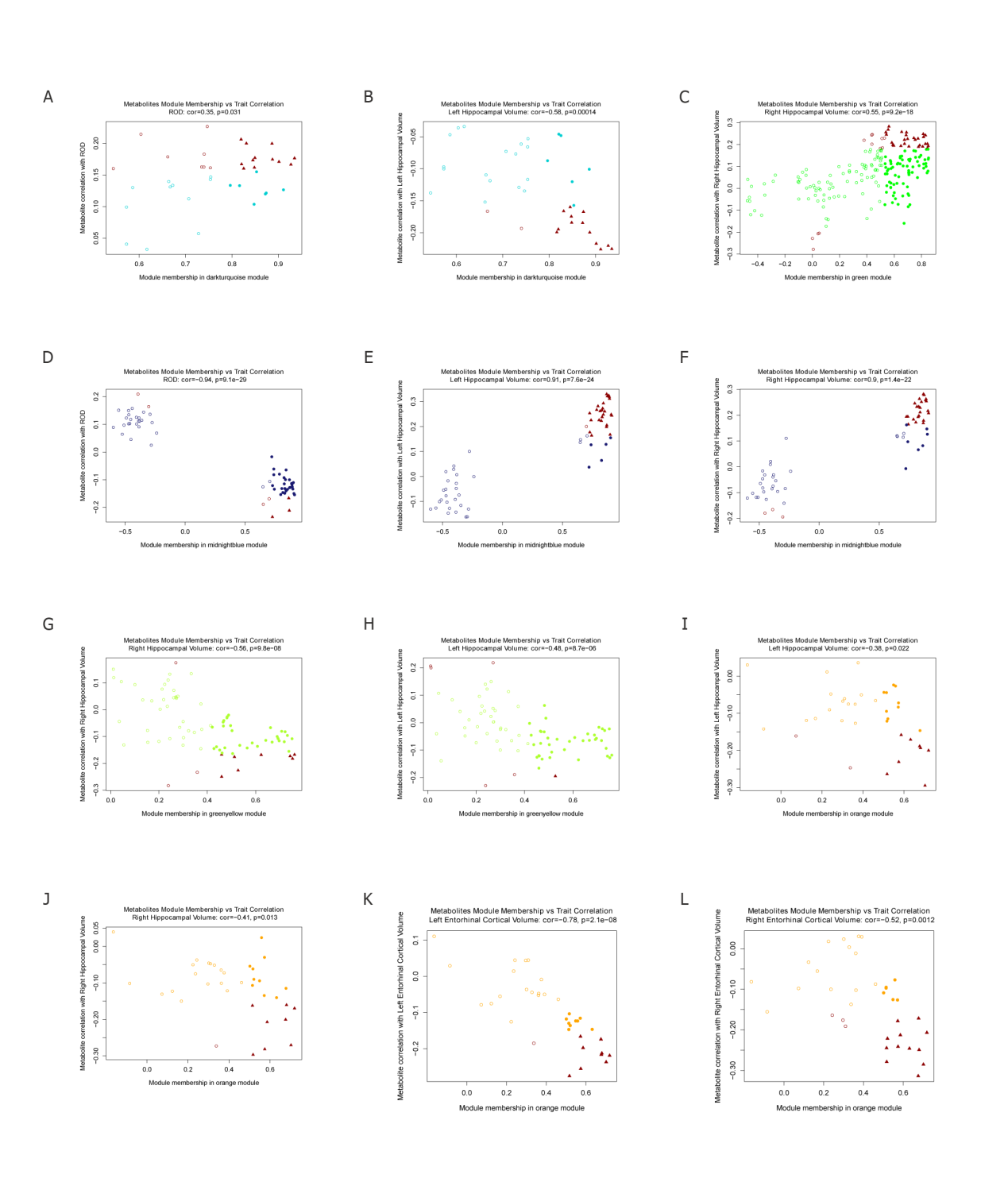


**Figure S8. Scatter plots of lipid module membership versus lipids-phenotypes correlation.** (A) kMEs in darkturquoise module vs. lipids-ROD; (B) kMEs in darkturquoise module vs. lipids-Hippocampal left volume; (C) kMEs in green module vs. lipids-Hippocampal right volume; (D) kMEs in midnightblue module vs. lipids-ROD; (E) kMEs in midnightblue module vs. lipids-Hippocampal left volume; (F) kMEs in midnightblue module vs. lipids-Hippocampal right volume; (G) kMEs in greenyellow module vs. lipids-Hippocampal right volume; (H) kMEs in greenyellow module vs. lipids-entorhinal cortex left volume; (I) kMEs in orange module vs. lipids-Hippocampal left volume; (J) kMEs in orange module vs. lipids-Hippocampal right volume; (K) kMEs in orange module vs. lipids-entorhinal cortex left volume; (L) kMEs in orange module vs. lipids-entorhinal cortex right volume.


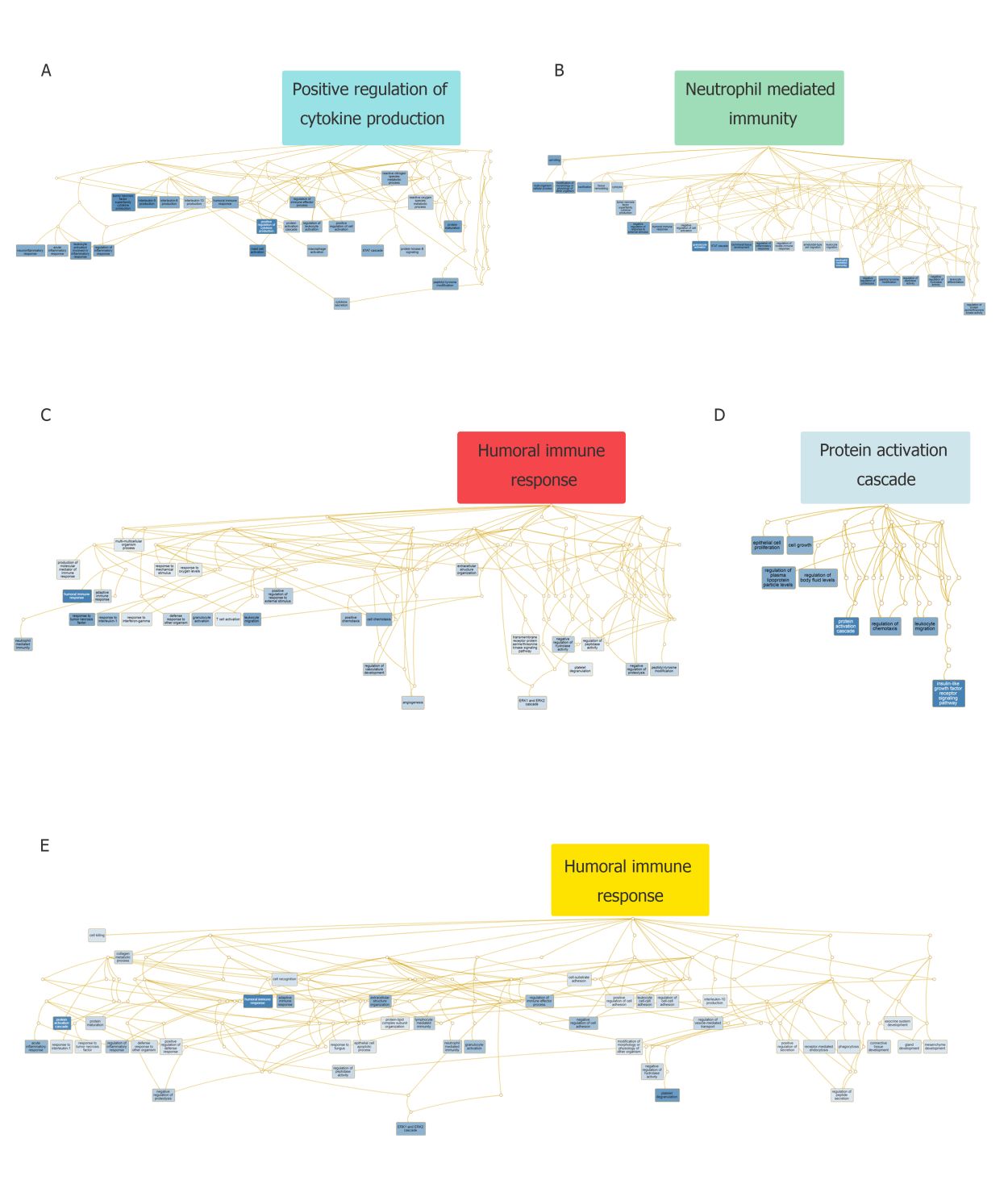


**Figure S9.** **DAGs summarize biological processes in five protein modules.** (A) cyan module; (B) lightgreen module; (C) lightcyan module; (D) red module; (E) yellow module. Each DAG includes the all biological processes passed the BH correction for each protein module. The top biological process for each module was listed in the box above the DAG, and the colour of the boxes corresponds to the colour of the module.


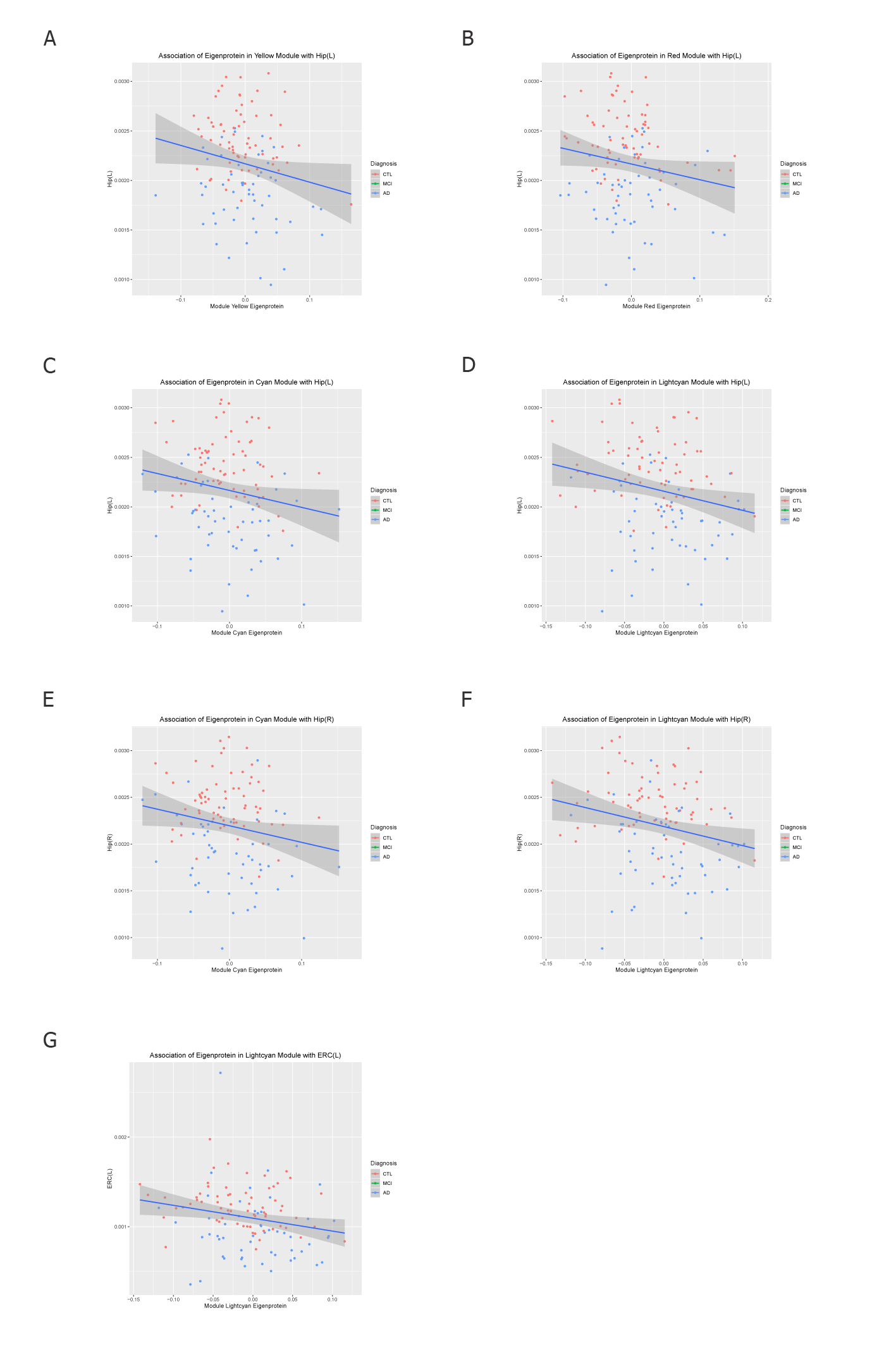


**Figure S10.** **Scatter plots of eigenproteins correlation with brain atrophy measures.** (A) Eigenprotein in yellow module vs. Hippocampal left volume ; (B) Eigenprotein in red module vs. Hippocampal left volume; (C) Eigenprotein in cyan module vs. Hippocampal left volume; (D) Eigenprotein in lightcyan module vs. Hippocampal left volume; (E) Eigenprotein in cyan module vs. Hippocampal right volume; (F) Eigenprotein in lightcyan module vs. Hippocampal right volume; (G) Eigenlipid in lightcyan module vs. entorhinal cortex left volume.
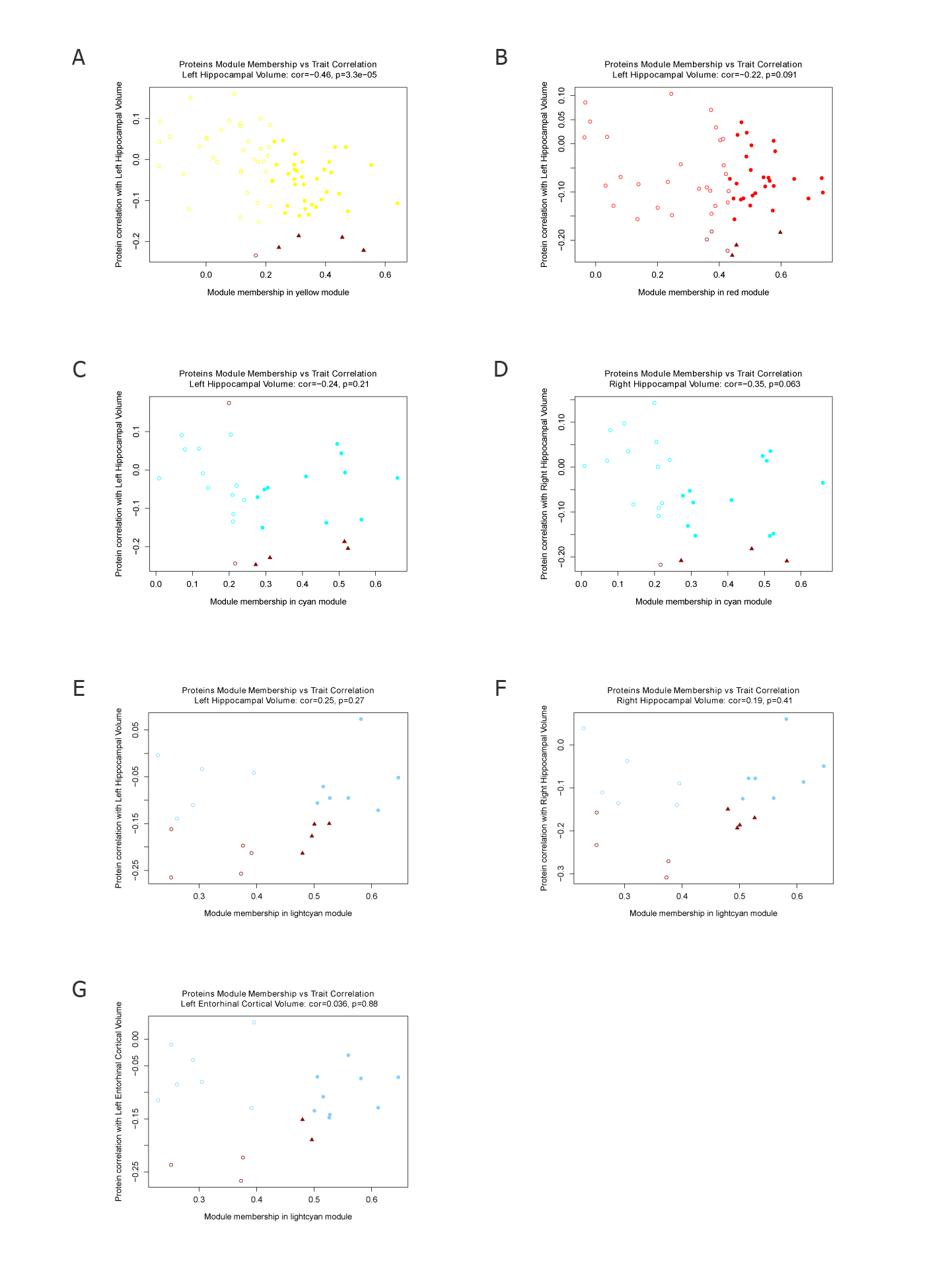


**Figure S11. Scatter plots of protein module membership versus proteins-phenotypes correlation.** (A) kMEs in yellow module vs. proteins-Hippocampal left volume; (B) kMEs in red module vs. proteins-Hippocampal left volume; (C) kMEs in cyan module vs. proteins-Hippocampal left volume; (D) kMEs in cyan module vs. proteins-Hippocampal right volume; (E) kMEs in lightcyan module vs. proteins-Hippocampal left volume; (F) kMEs in lightcyan module vs. proteins-Hippocampal right volume; (G) kMEs in lightcyan module vs. proteins-entorhinal cortex left volume.


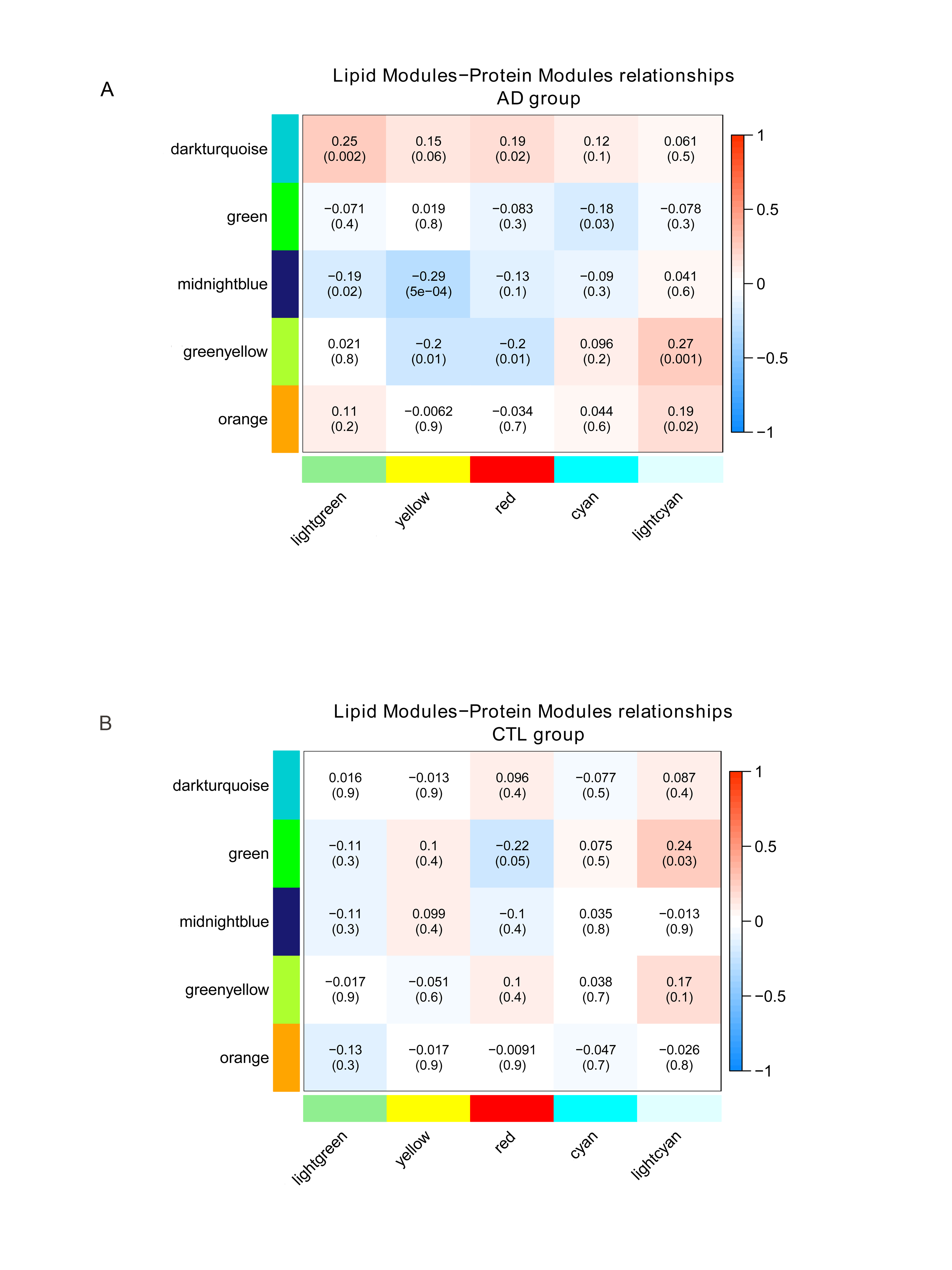


**Figure S12. Scatter plots of protein module membership versus proteins-phenotypes correlation.** Heat map showing the Pearson correlations and p values (in bracket) between 5 lipid modules (rows) associated with phenotypes and 5 protein modules (columns) associated with phenotypes in (A) AD group and (B) CTL group.


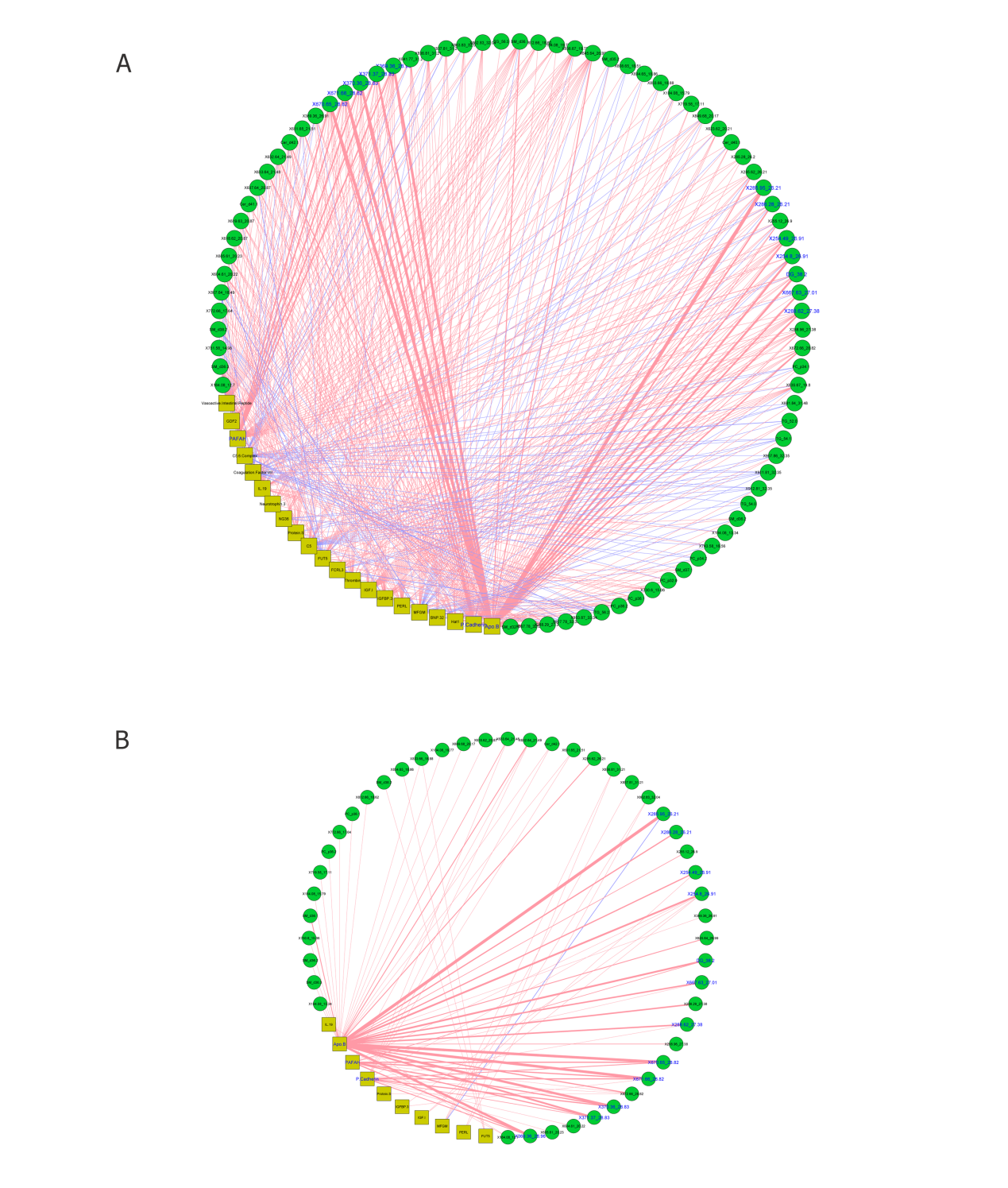


**Figure S13. Correlation networks for lipid greenyellow module and protein lightcyan module.** (A) Correlation between lipids and proteins within the range of (-1, -0.1) & (0.1, 1); (B) Correlation between lipids and proteins within the range of (-1, -0.2) & (0.2, 1). Pearson correlation was applied. Green circles represent lipids and yellow squares represent proteins. Pink edge mean positive correlation and blue edge means negative correlation. The width of the edge correlates with the strength of the correlation. Lipids/protein names were labelled in blue if correlation p values passing Holm–Bonferroni correction.


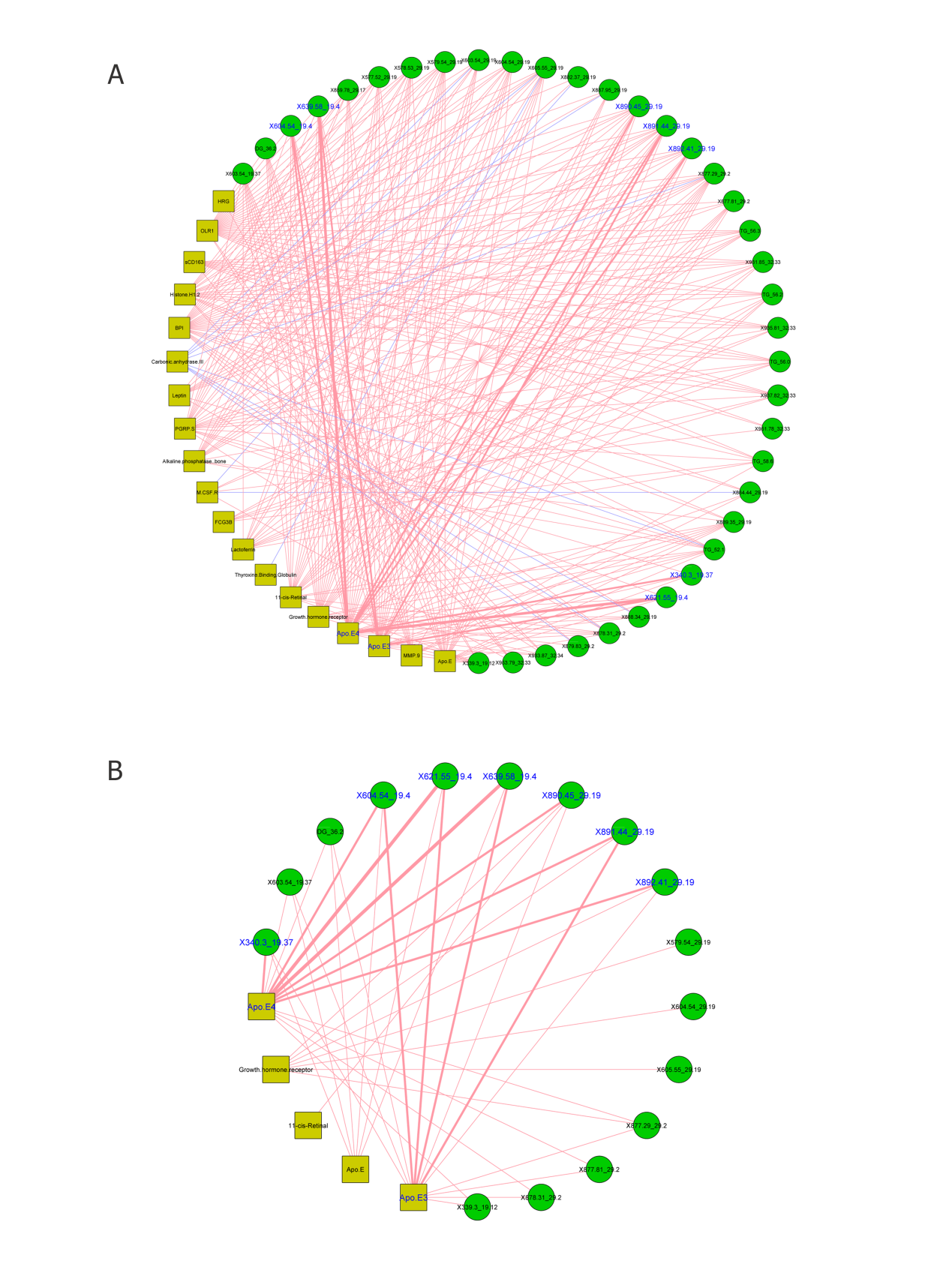


**Figure S14. Correlation networks for lipid darkturquoise module and protein lightgreen module.** (A) Correlation between lipids and proteins within the range of (-1, -0.1) & (0.1, 1); (B) Correlation between lipids and proteins within the range of (-1, -0.2) & (0.2, 1). Pearson correlation was applied. Green circles represent lipids and yellow squares represent proteins. Pink edge mean positive correlation and blue edge means negative correlation. The width of the edge correlates with the strength of the correlation. Lipids/protein names were labelled in blue if correlation p values passing Holm–Bonferroni correction.

References

[1] I. E. Jansen *et al.*, ‘Genome-wide meta-analysis identifies new loci and functional pathways influencing Alzheimer’s disease risk’, *Nat. Genet.*, vol. 51, no. 3, pp. 404–413, 2019, doi: 10.1038/s41588-018-0311-9.

[2] B. W. Kunkle *et al.*, ‘Genetic meta-analysis of diagnosed Alzheimer’s disease identifies new risk loci and implicates A beta, tau, immunity and lipid processing (vol 51, pg 414, 2019)’, *Nat. Genet.*, vol. 51, no. 9, pp. 1423–1424, Sep. 2019, doi: 10.1038/s41588-019-0495-7.

[3] S. Moreno-Grau *et al.*, ‘Genome-wide association analysis of dementia and its clinical endophenotypes reveal novel loci associated with Alzheimer’s disease and three causality networks: The GR@ACE project’, *Alzheimers Dement.*, vol. 15, no. 10, pp. 1333–1347, Oct. 2019, doi: 10.1016/j.jalz.2019.06.4950.

[4] J. C. Lambert *et al.*, ‘Meta-analysis of 74,046 individuals identifies 11 new susceptibility loci for Alzheimer’s disease’, *Nat Genet*, vol. 45, no. 12, pp. 1452–8, Dec. 2013, doi: 10.1038/ng.2802.

[5] R. E. Marioni *et al.*, ‘GWAS on family history of Alzheimer’s disease’, *Transl. Psychiatry*, vol. 8, no. 1, Art. no. 1, May 2018, doi: 10.1038/s41398-018-0150-6.
